# Prediction Accuracy in Multivariate Repeated-Measures Bayesian Forecasting Models with Examples Drawn from Research on Sleep and Circadian Rhythms

**DOI:** 10.1155/2016/4724395

**Published:** 2016-01-14

**Authors:** Clark Kogan, Leonid Kalachev, Hans P. A. Van Dongen

**Affiliations:** ^1^Sleep and Performance Research Center, Washington State University, Spokane, WA 99210, USA; ^2^Department of Mathematical Sciences, University of Montana, Missoula, MT 59812, USA; ^3^Elson S. Floyd College of Medicine, Washington State University, Spokane, WA 99210, USA

## Abstract

In study designs with repeated measures for multiple subjects, population models capturing within- and between-subjects variances enable efficient individualized prediction of outcome measures (response variables) by incorporating individuals response data through Bayesian forecasting. When measurement constraints preclude reasonable levels of prediction accuracy, additional (secondary) response variables measured alongside the primary response may help to increase prediction accuracy. We investigate this for the case of substantial between-subjects correlation between primary and secondary response variables, assuming negligible within-subjects correlation. We show how to determine the accuracy of primary response predictions as a function of secondary response observations. Given measurement costs for primary and secondary variables, we determine the number of observations that produces, with minimal cost, a fixed average prediction accuracy for a model of subject means. We illustrate this with estimation of subject-specific sleep parameters using polysomnography and wrist actigraphy. We also consider prediction accuracy in an example time-dependent, linear model and derive equations for the optimal timing of measurements to achieve, on average, the best prediction accuracy. Finally, we examine an example involving a circadian rhythm model and show numerically that secondary variables can improve individualized predictions in this time-dependent nonlinear model as well.

## 1. Introduction

Significant steps forward in the analysis of repeated-measures data were made with the introduction of linear and nonlinear mixed-effects models [1–3], which distinguish within-subjects variance (from multiple measurements in each subject) versus between-subjects variance (from multiple subjects being measured). Distinguishing these types of variance can also be thought of as explicitly modeling random error in the data. This can be useful in understanding how different individuals are from one another as compared to how different multiple measurements are for given individuals. In research on sleep and sleepiness, for example, breakthroughs made possible by mixed-effects models include elucidation of the dose-response effects of sustained sleep restriction on sleep architecture and neurobehavioral impairment [4, 5] and demonstration of the trait characteristics of individual differences in vulnerability to sleep loss [6]. In recent years, mixed-effects model approaches to statistical regression and analysis of variance have become widely available in statistical software packages. They are nowadays the methodology of choice for many repeated-measures investigations in sleep research and other fields of study.

A further advance was the introduction of a model individualization technique called Bayesian posterior distribution estimation or Bayesian forecasting. This technique was first used in sleep research to overcome a shortcoming of biomathematical models of fatigue and performance. Existing models did not account for individual differences in sleep regulation and vulnerability to sleep loss and therefore did not accurately predict performance for given individuals. Bayesian forecasting addressed this shortcoming by utilizing the separation of within- and between-subjects variance in model parameters as enabled by mixed-effects modeling [2]. In Bayesian forecasting, the between-subjects variance of model parameters serves as Bayesian prior information. Measurements from a new individual, not previously studied, are combined with the prior information to efficiently derive model parameters tailored to the new individual, thereby yielding a subject-specific mathematical model [2, 7, 8]. As a bonus, the Bayesian forecasting technique also yields quantitative estimates of the accuracy of individualized predictions made with the subject-specific mathematical model [9].

**Figure 1:**
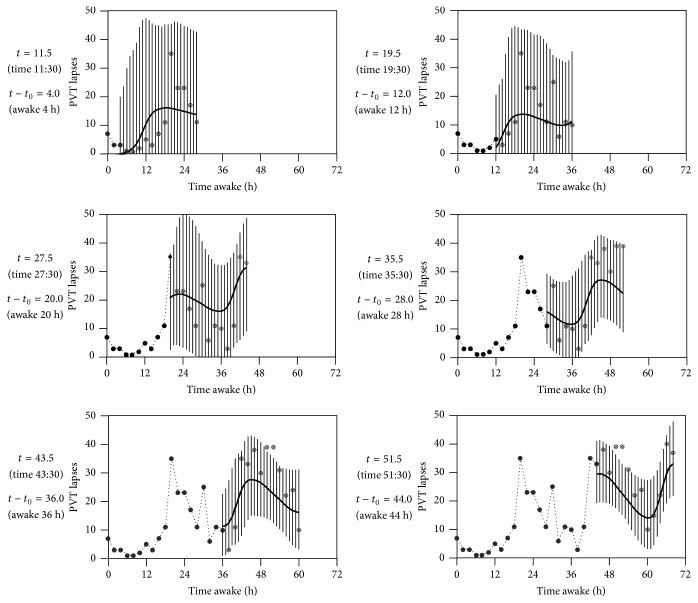
Lapses of attention on a psychomotor vigilance test (PVT) for a subject in a study involving 88 h of total sleep deprivation under controlled laboratory conditions. In each of the six plots, different amounts of subject data are assumed known (black dots), and the Bayesian forecasting procedure is applied to the known data to construct predictions of PVT number of lapses for a 24 h interval immediately following the most recent collected data point at time *t*. For the 24 h interval, 95% prediction intervals (vertical bars) are shown, along with the remainder of the data for comparison (gray dots). The beginning of the 88 h sleep deprivation period, *t*_0_, was at 07:30. Graphs taken from [7] with permission.

In a published example, Bayesian forecasting was implemented for the two-process model of sleep regulation [10, 11] to predict performance impairment of selected individuals undergoing a period of total sleep deprivation (see Figure 1). Comparisons with the individuals' actual data revealed that the model parameters converged efficiently to those that best characterized each individual, and the response predictions were significantly more accurate than could have been achieved with the original, nonindividualized two-process model [7].

In Bayesian forecasting, individualized parameter estimates are derived from the posterior distributions of the parameters in question after combining prior distributions with the measurement(s) from the individual, and individualized model predictions for future outcomes or responses are obtained following estimation of the posterior distribution of the expected responses at given times. A variety of methods are available for obtaining parameter estimates and response predictions once posterior distributions have been estimated. We employ the Bayesian mean squared error (BMSE) [12] and make use of the Bayesian minimum mean squared error (MMSE) estimator [13, 14], which produces unbiased point estimates that minimize the BMSE [15]. The BMSE of parameter estimates and response predictions is dependent on the amount of data available for the individual at hand and the magnitude of between-subjects variance captured in the Bayesian prior distributions.

In cases where between-subjects variance is relatively large, such as performance responses to sleep loss [6, 16], measurement data for the individual at hand are more critical for making accurate individualized response predictions. Sparseness of such measurement data (e.g., due to practical or cost-based limitations) can result in unacceptably low levels of accuracy. For example, Bayesian forecasting could be used to develop a drowsy driver warning system based on a mathematical model of fatigue and performance [17, 18] calibrated to predict lateral lane deviation, using camera-based measurements of lane position to individualize the model for the driver. However, when lane markers are covered with snow and cameras are unable to determine vehicle position relative to the lane, the individualization effort becomes less effective.

To address this limitation, we consider the use of secondary variables to increase data availability, boost response prediction accuracy, and/or reduce data collection costs for individualized response prediction. For example, in the case of a drowsy driver warning system, camera-based measurements of lateral lane deviation serving as the primary response variable could be augmented with in-car secondary variables such as steering wheel variability or driver eyelid closure assessments. However, individualization of predictions based on two or more measurement variables would only be straightforward if individual differences in responses on these variables are equivalent (cf. [19]). Generally, this is not what the evidence shows. As a case in point, trait individual differences in vulnerability to performance impairment due to sleep loss vary considerably across outcome variables [6, 20], such that the most vulnerable individuals based on one variable are not necessarily also the most vulnerable individuals based on another variable. Therefore, when considering two or more response variables as the basis for individualized prediction, it is essential to account statistically for the level of congruence between the response variables.

Here we develop a multivariate statistical framework for individualized prediction of sleep or performance variables, based on Bayesian forecasting with measurements of a primary response variable, augmented with one or more measurements of secondary response variables. The response variables are assumed to follow equivalent dynamics over time, such that they can be described by the same model framework after appropriate scaling. This is a reasonable assumption in the case of, for example, models describing sleep variables measured repeatedly across multiple nights [21] and models describing performance changes over time in response to sleep loss [22]. We make use of multivariate Bayesian prior distributions of the primary and secondary variables, assumed to have been assessed in advance by means of mixed-effects modeling [2] or other suitable techniques. The between-subjects correlation(s) between the primary and secondary variables, used here to account for the level of congruence between the response variables, is assumed to have been estimated as part of the covariance matrix of the multivariate Bayesian prior distributions. The between-subjects correlation(s) are assumed to be at least moderately strong, lest the secondary variables contain essentially no information about the primary response variable that rises above the level of measurement noise.

Our focus in this paper is on prediction accuracy in the multivariate Bayesian forecasting technique. We develop the technique by first considering the details of making individualized response predictions and estimating their accuracy for a simple univariate intercept model. To demonstrate how secondary, correlated responses can be used to make more accurate individualized predictions, we expand the intercept model to include both primary and secondary responses. Assuming fixed costs of data collection for each of the responses, we show how to optimize data collection given a desired level of accuracy for predictions of the primary response variable.

We then consider a linear approximation of the homeostatic component of the two-process model [11] and derive a closed form equation of the BMSE to quantify prediction accuracy in this time-dependent model. For this example, we study the problem of optimizing the timing of measurements in order to enhance the individualized prediction accuracy most efficiently. Finally, we consider more complicated bivariate models, both linear and nonlinear, for which the BMSE cannot be determined in closed form. For these models, we describe a process of numerically assessing the BMSE for individualized prediction based on a primary response variable in Bayesian forecasting augmented with measurements of a secondary response.

## 2. Subject-Specific Bayesian Models

First we discuss a modeling framework for Bayesian forecasting. Consider a response variable *y*, dependent on a subject-specific trait parameter *θ*. Let *i* be an index for individual, ranging from 1 to *N*, and let *j* be an index for observations ordered by time, nested within individual. Suppose that the response variable can be modeled by

(1) $$\begin{array}{c} y_{ij}=f\left(\;t_{ij},\theta_{i}\;\right)+\epsilon_{ij}, \end{array}$$

where *f*(*t*_*ij*_, *θ*_*i*_) represents the model function, *t*_*ij*_ represents a fixed measurement time, *θ*_*i*_ represents a random subject-specific parameter, and *ϵ*_*ij*_ represents additive measurement error. We assume that the distributions for *θ*_*i*_ and *ϵ*_*ij*_ are known. Equation (1) and the distributions of *θ*_*i*_ and *ϵ*_*ij*_ constitute a population model.

Limiting our focus to a particular individual, we may remove the subscript *i* and model the subject's responses as

(2) $$\begin{array}{c} y_{j}=f_{j}+\epsilon_{j}, \end{array}$$

where *f*_*j*_ is used to denote *f*(*t*_*j*_, *θ*).

Suppose that a total of *m* responses (*y*_1_,…, *y*_*m*_) have been measured for the individual at hand, and let *j* ^*∗*^ denote the index of a response *y*_*j*^*∗*^_ at some future time *t*_*j*^*∗*^_. We consider a prediction (estimator) of the expected response *E*[*y*_*j*^*∗*^_∣*θ*] = *f*_*j*^*∗*^_, which we denote as ${\hat{f}}_{j^{*}}$. Our interest is in constructing ${\hat{f}}_{j^{*}}$ such that the expected accuracy for an arbitrary, given individual from the population is minimized.

We define the accuracy using the squared error ${\left(f_{j^{*}}-{\hat{f}}_{j^{*}}\right)}^{2}$. The expected accuracy, which is referred to as the Bayesian mean squared error (BMSE), is thus given by

(3) $$\begin{array}{c} M_{{\hat{f}}_{j^{*}}}\equiv BMSE\left(\;{\hat{f}}_{j^{*}}\;\right)\equiv E\left[\;{\left(\;f_{j^{*}}-{\hat{f}}_{j^{*}}\;\right)}^{2}\;\right], \end{array}$$

where the expectation is taken with respect to the marginal probability density function (pdf) of *y*_1_,…, *y*_*m*_ and *θ*.

We refer to the prediction that minimizes the BMSE as a minimum mean squared error (MMSE) prediction. After observing data from a particular individual, *y*_1_,…, *y*_*m*_, and constructing the MMSE prediction ${\hat{f}}_{j^{*}}$, we seek to assess the expected accuracy of this particular prediction. This can be done with confidence intervals on *f*_*j*^*∗*^_, obtained from quantiles of the posterior distribution of *θ*∣*y*_1_,…, *y*_*m*_. In the following sections, we describe specific types of models and investigate the BMSE and the MMSE that minimizes it.

## 3. Univariate Random Intercept Model

We consider a random intercept model obtained from (2) by letting *f*_*j*_ = *θ*:

(4) yj=θ+ϵj,

(5) θ~Nμ,δ2,

(6) ϵj~N0,σ2,

where *j* = 1,…, *m*. For a particular individual, the expected response at time *t*_*j*^*∗*^_ is

(7) $$\begin{array}{c} E\left[\;y_{j^{*}}\;|\;\theta \;\right]=f_{j^{*}}=\theta . \end{array}$$

It follows that the MMSE prediction of *E*[*y*_*y*^*∗*^_∣*θ*] is

(8) $$\begin{array}{c} {\hat{f}}_{j^{*}}=\hat{\theta }, \end{array}$$

where $\hat{\theta }$ is the MMSE of *θ*. The estimator $\hat{\theta }$ (and therefore ${\hat{f}}_{j^{*}}$) is given by [23]:

(9) $$\begin{array}{c} \hat{\theta }=\upsilon \bar{y}+\left(\;1-\upsilon \;\right)\mu , \end{array}$$

where $\bar{y}$ is the sample mean of the measured responses and

(10) $$\begin{array}{c} \upsilon =\frac{\delta^{2}}{\delta^{2}+\sigma^{2}/m}. \end{array}$$

The variance of the posterior distribution of *θ*∣*y*_1_,…, *y*_*m*_ (and therefore ${\hat{f}}_{j^{*}}|y_{1},\ldots ,y_{m}$) is [23]:

(11) $$\begin{array}{c} Var\left(\;\theta \;|\;y_{1},\ldots ,y_{m}\;\right)=\frac{\sigma^{2}}{m}\left(\;\frac{\delta^{2}}{\delta^{2}+\sigma^{2}/m}\;\right). \end{array}$$

Furthermore, the BMSE of $\hat{\theta }$ (and therefore ${\hat{f}}_{j^{*}}$) is given by [23]:

(12) $$\begin{array}{c} M_{\hat{\theta }}=Var\left(\;\theta \;|\;y_{1},\ldots ,y_{m}\;\right). \end{array}$$

The MMSE prediction ${\hat{f}}_{j^{*}}$ (given by (8) and (9)) represents a trade-off between knowledge about the population and knowledge about the subject at hand. This trade-off is embodied by the weighting factor *υ* in (10). When no data are available for the subject at hand, *υ* = 0, resulting in a prediction made at ${\hat{f}}_{j^{*}}=\mu$, where *μ* is, in this case, representative of the population mean *E*[*θ*] as well as the population mean response *E*[*y*_*j*^*∗*^_]:

(13) $$\begin{array}{c} E\left[\;y_{j^{*}}\;\right]=E\left[\;\theta \;\right]=\mu . \end{array}$$

As we begin to collect* subject-specific* data, the weighting factor moves towards the value *υ* = 1, resulting in a prediction that converges to the subject-specific sample mean as data collection continues.

Likewise, when no data are available for the subject at hand, the expected accuracy of the prediction is $M_{{\hat{f}}_{j^{*}}}=\delta^{2}$. The term *δ* ^2^ is, in this case, representative of the population variance as well as the variance in mean response over the population:

(14) VarEyj∗ ∣ θ=EEyj∗ ∣ θ−EEyj∗ ∣ θ2=EEyj∗ ∣ θ−Eyj∗2=Eθ−μ2=δ2,

where the expectation *E*[*y*_*j*^*∗*^_∣*θ*] is evaluated with respect to the conditional distribution of *y*_*j*^*∗*^_∣*θ*, and all other expectations are evaluated with respect to the marginal distribution of *y*_*j*^*∗*^_. Per (12), as we begin to collect subject-specific data, the expected accuracy of the prediction improves (i.e., the BMSE decreases, where smaller is better).

**Figure 2:**
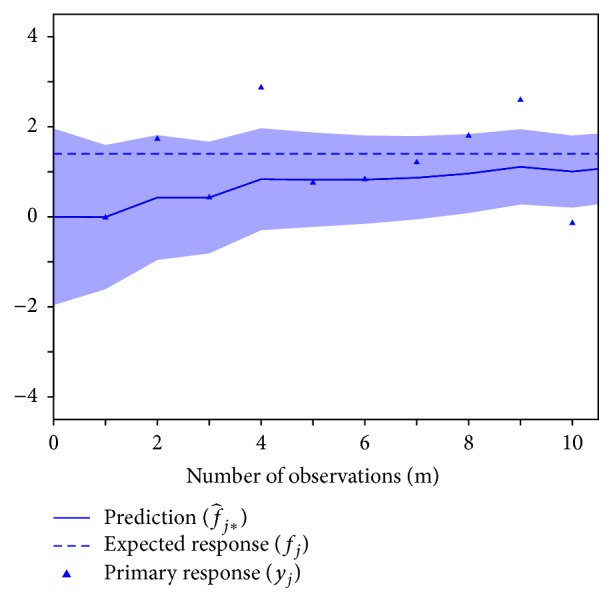
MMSE predictions of the expected response ${\hat{f}}_{j^{*}}$ from the univariate random intercept model specified in (4), and corresponding 95% confidence intervals, determined by incorporating simulated observations *y*_*j*_ iteratively. The individualized Bayesian forecasting predictions begin at the population mean expected response *μ* = 0 when no individual data are used and converge to the subject's expected individualized response *f*_*j*^*∗*^_ = *θ* = 1.4 as more subject-specific data are collected.

To illustrate how predictions in this model depend on the amount of subject-specific data collected, we conducted a simulation of model (4) for a particular individual. For this example, the parameter *θ* for the individual was chosen far from the population mean (when compared to the magnitude of the population variance), in order to make the transition from the population mean to the true expected response *f*_*j*^*∗*^_ large enough so as not to be obscured by measurement noise in the example. We assumed the population parameters *μ* = 0 and *δ* = 1 and chose the subject-specific parameter value *θ* = 1.4. We simulated *m* = 10 data points for the individual, with a standard deviation of measurement error of *σ* = 1. The MMSE prediction ${\hat{f}}_{j^{*}}$ was calculated by incorporating observations *y*_*j*_ iteratively.  Figure 2 shows ${\hat{f}}_{j^{*}}$ plotted against the number of data points used. The variance of the posterior distribution for *f*_*j*^*∗*^_ from (11) was used to construct confidence intervals on *f*_*j*^*∗*^_. As expected, for the individual considered, the prediction ${\hat{f}}_{j^{*}}$ began at the population mean and moved to the true expected response with shrinking confidence interval as more simulated data were collected.

## 4. Bivariate Subject-Specific Bayesian Models

When subject-specific data are sparse, individualized predictions may not, on average, reach acceptable levels of accuracy. Accuracy may be improved by including data from a secondary subject-specific data source. However, individual differences in one response variable may not be identical to individual differences in another (e.g., [6, 21]). Therefore, data from a secondary response variable may not simply be used as a substitute for the primary response variable. Rather, to improve prediction accuracy on the primary response by incorporating data from the secondary response, the between-subjects correlation between the primary and secondary response variables must be taken into account.

Here we derive the average accuracy of individualized predictions of a primary response variable based on distinct primary and secondary subject-specific response variables, accounting for between-subjects correlation between the two responses. Let *i* be an index for individual, let *r* be an index for response type, and let *j* be an index for observations ordered by time, nested within individual and response type. Suppose that the response *y*_*rij*_ can be modeled by

(15) $$\begin{array}{c} y_{rij}=f\left(\;t_{rij},\theta_{ri}\;\right)+\epsilon_{rij}, \end{array}$$

where *f*(*t*_*rij*_, *θ*_*ri*_) represents the model function, *t*_*rij*_ represents measurement time, *θ*_*ri*_ represents a random subject-specific parameter associated with response type *r*, and *ϵ*_*rij*_ represents additive measurement error. Limiting our focus to a particular individual, we may remove the subscript *i* and model the subject's responses as

(16) $$\begin{array}{c} y_{rj}=f_{rj}+\epsilon_{rj}, \end{array}$$

where *f*_*rj*_ is used to denote *f*(*t*_*rj*_, *θ*_*r*_). Suppose that a total of *m*_*r*_ responses have been observed for each response type *r* for the individual at hand. In the next sections, we focus on constructing the MMSE prediction for the expected primary response, ${\hat{f}}_{1j^{*}}$.

## 5. Bivariate Random Intercept Model

We consider the bivariate random intercept model obtained from (16) by letting *f*_*rj*_ = *θ*_*r*_:

(17) $$\begin{array}{c} y_{rj}=\theta_{r}+\epsilon_{rj}, \end{array}$$

where *r* = 1,2. The scalar model can be converted to vector form by concatenating the responses for each response type,

(18) y1=y11⋮y1m1,y2=y21⋮y2m2,

and then concatenating the response vectors of different types,

(19) $$\begin{array}{c} y=\left(\begin{array}{c} y_{1}\\ y_{2} \end{array}\right). \end{array}$$

A similar assembly of the measurement errors can be done so that

(20) ϵ1=ϵ11⋮ϵ1m1,ϵ2=ϵ21⋮ϵ2m2,ϵ=ϵ1ϵ2.

An assembly of the parameters can be accomplished by first constructing the parameter vector,

(21) $$\begin{array}{c} \theta =\left(\begin{array}{c} \theta_{1}\\ \theta_{2} \end{array}\right), \end{array}$$

and the design matrix,

(22) $$\begin{array}{c} H=\left(\begin{array}{cc} 1 & 0\\ \vdots & \vdots \\ 1 & 0\\ 0 & 1\\ \vdots & \vdots \\ 0 & 1 \end{array}\right), \end{array}$$

so that the single individual model of (17) can be vectorized as

(23) $$\begin{array}{c} y=H\theta +\epsilon . \end{array}$$

We consider the case where subject-specific traits and measurement errors are both normally distributed,

(24) θ~Nμ,Cθ,

(25) ϵ~N0,Cϵ,

where ***μ***, **C**_*θ*_, and **C**_*ϵ*_ are fixed population characteristics.

Correlations between primary and secondary response variables **y**_1_ and **y**_2_ can be modeled as arising from a correlation between *θ*_1_ and *θ*_2_ (between-subjects correlation) or correlations between **ϵ**_1_ and **ϵ**_2_ (within-subjects correlation). Here, we assume that response correlations arise from between-subjects correlations:

(26) $$\begin{array}{c} C_{\theta }=\left(\begin{array}{cc} \delta_{1}^{2} & \rho \delta_{1}\delta_{2}\\ \rho \delta_{1}\delta_{2} & \delta_{2}^{2} \end{array}\right), \end{array}$$

where *ρ* (−1 < *ρ* < 1) represents the between-subjects correlation between primary and secondary response variable means and *δ*_*r*_^2^ represents the between-subjects variance for response variable *r*. Furthermore, we assume that measurement errors are uncorrelated with response variable-specific variance *σ*_*r*_^2^, so that correlations between the response variables arise only from the between-subjects components. For subject-specific repeated-measures data with no covariates for two response variables, it may be fair to consider the error variance within subjects to be independent as long as perturbations from the intercepts do not tend to be common over both response types.

The error covariance matrix for response type *r* is a diagonal matrix with dimension *m*_*r*_, where each nonzero element is the type-specific error variance *σ*_*r*_^2^,

(27) $$\begin{array}{c} \Sigma_{r}=\left(\begin{array}{ccc} \sigma_{r}^{2} & 0 & \ldots \\ 0 & \sigma_{r}^{2} & \ddots \\ \vdots & \ddots & \ddots \end{array}\right). \end{array}$$

The full error covariance matrix can then be built from the type-specific blocks,

(28) $$\begin{array}{c} C_{\epsilon }=\left(\begin{array}{cc} \Sigma_{1} & 0\\ 0 & \Sigma_{2} \end{array}\right). \end{array}$$

The bivariate Bayesian model we consider here is fully characterized by (23)–(28).

As was the case for model (4), for a particular individual, the expected primary response at time *t*_1*j*^*∗*^_ is

(29) $$\begin{array}{c} E\left[\;y_{1j^{*}}\;|\;\theta \;\right]=f_{1j^{*}}=\theta_{1}. \end{array}$$

The MMSE prediction is

(30) $$\begin{array}{c} {\hat{f}}_{1j^{*}}={\hat{\theta }}_{1}. \end{array}$$

The MMSE estimator for model (23) is [23]:

(31) $$\begin{array}{c} \hat{\theta }=\mu +{\left(\;C_{\theta }^{-1}+H^{\prime }C_{\epsilon }^{-1}H\;\right)}^{-1}H^{\prime }C_{\epsilon }^{-1}\left(\;y-H\mu \;\right). \end{array}$$

The MMSE estimator ${\hat{\theta }}_{1}$ can be extracted as the first element of $\hat{\theta }$.

The variance of the posterior distribution of ***θ***∣**y** is given by

(32) $$\begin{array}{c} Var\left(\;\theta \;|\;y\;\right)={\left(\;C_{\theta }^{-1}+H^{\prime }C_{\epsilon }^{-1}H\;\right)}^{-1}. \end{array}$$

The variance of the posterior distribution of *θ*_1_∣**y** (and therefore *f*_1*j*^*∗*^_∣**y**) can be obtained by extracting the first element from the diagonal of ***θ***∣**y**. The BMSE of the MMSE estimator ***θ*** can be obtained from the parameter BMSE matrix $M_{\hat{\theta }}$, which is given by [23]:

(33) $$\begin{array}{c} M_{\hat{\theta }}=Var\left(\;\theta \;|\;y\;\right). \end{array}$$

The BMSE of ${\hat{\theta }}_{1}$ (and therefore ${\hat{f}}_{1j^{*}}$) can be obtained by extracting the first element from the diagonal of $M_{\hat{\theta }}$. Substituting (22), (26) and (28) into (33), we find that the BMSE for ${\hat{\theta }}_{1}$ can be simplified as follows:

(34) $$\begin{array}{c} M_{{\hat{\theta }}_{1}}={\left(\;\frac{m_{1}}{\sigma_{1}^{2}}+\frac{1}{\delta_{1}^{2}}+\lambda \left(\;m_{2}\;\right)\;\right)}^{-1}, \end{array}$$

where

(35) $$\begin{array}{c} \lambda \left(\;m_{2}\;\right)=\frac{\rho^{2}\delta_{2}^{2}/\delta_{1}^{2}}{\delta_{2}^{2}\left(1-\rho^{2}\right)+\sigma_{2}^{2}/m_{2}}. \end{array}$$

**Figure 3:**
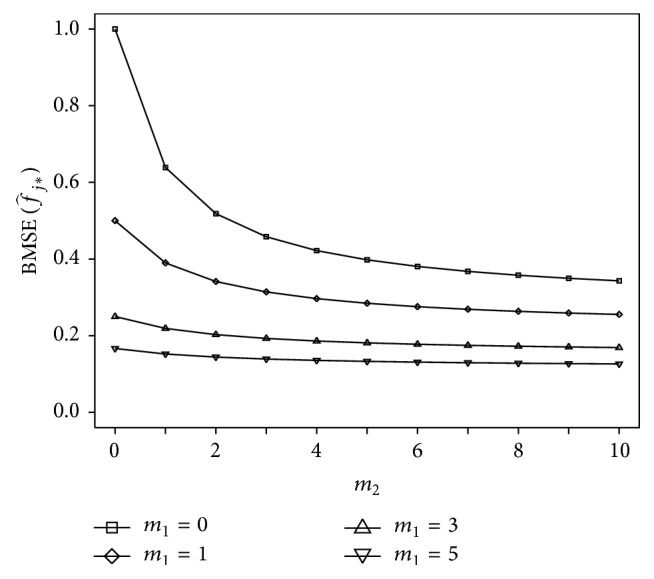
BMSE (i.e., accuracy) of MMSE predictions of the expected primary response ${\hat{f}}_{j^{*}}$ for the bivariate random intercept model specified in (23), as a function of the amount of data collected on a secondary response variable (*m*_2_), shown for different amounts of data collected on the primary response variable (*m*_1_). The figure illustrates that the accuracy of individualized Bayesian forecasting predictions improves progressively with just a few measurements of the secondary response variable when measurements of the primary response variable are increasingly sparse.

Figure 3 illustrates the dependence of the BMSE of ${\hat{f}}_{j^{*}}$ on the number of observations from the primary and secondary responses. For this illustration, the population parameters were fixed at the values *δ*_1_ = 1, *δ*_2_ = 1, *ρ* = 0.85, *σ*_1_ = 1, and *σ*_2_ = 1. The figure shows the decrease of the BMSE as a function of the collection of secondary response measurements, given different numbers of primary response measurements. For a large number of primary response measurements, little change in BMSE is derived from the secondary response. However, for a small number of primary response measurements, the BMSE decreases substantially with just a few measurements of the secondary response.

**Figure 4:**
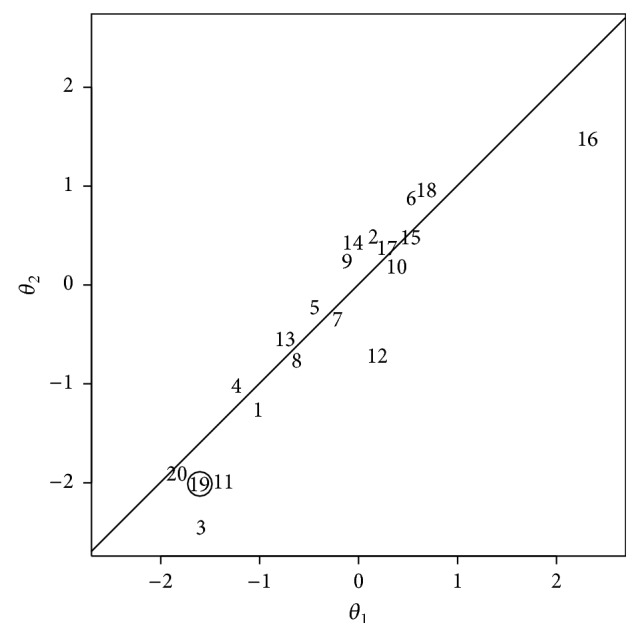
Subject-specific parameter pairs, simulated from a probability distribution specified by (24), from which a simulated individual was selected for a bivariate Bayesian forecasting simulation. Each number represents the parameter pair ***θ*** = (*θ*_1_, *θ*_2_) for a different simulated individual. The circled individual (#19) is used for the illustration in Figure 5. The diagonal line shows where the points would fall given a between-subjects correlation of *ρ* = 1.

To further illustrate the bivariate Bayesian forecasting procedure, simulated subject-specific parameter pairs ***θ*** = (*θ*_1_, *θ*_2_) from the population distribution given by (24) with *δ*_1_ = 1, *δ*_2_ = 1, and *ρ* = 0.9 were generated for *N* = 20 individuals, as shown in Figure 4. From this simulated set, individual #19 (circled in Figure 4) with subject-specific parameter vector ***θ*** = (−1.6; −2.0) was chosen to illustrate the transition of the primary response prediction from the population mean response to the true expected response *f*_1*j*^*∗*^_ through Bayesian forecasting. For this individual, we simulated errors from (25) using $\sigma_{1}=\sqrt{2}$, $\sigma_{2}=\sqrt{0.5}$, *m*_1_ = 10, and *m*_2_ = 10. Bivariate responses were then constructed using (23).

**Figure 5:**
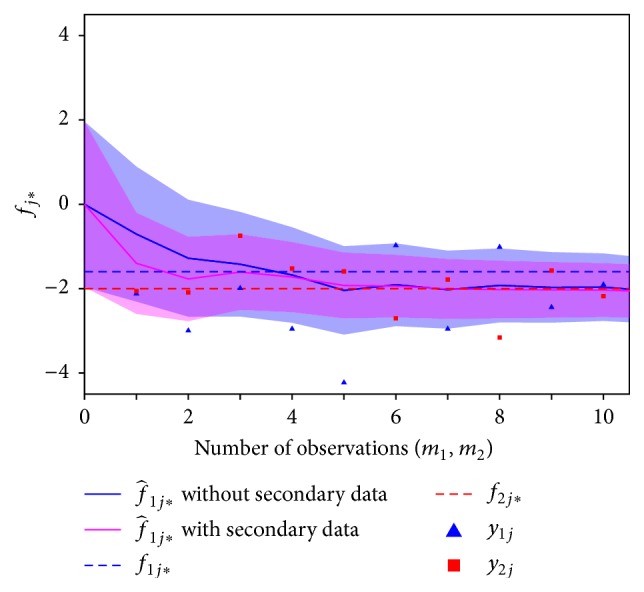
MMSE predictions of the expected primary response ${\hat{f}}_{j^{*}}$ from the bivariate random intercept model specified in (23), and corresponding 95% confidence intervals (shaded areas) for individual #19. The MMSE estimator ${\hat{f}}_{j^{*}}$ was iteratively determined assuming only primary responses were observed, as well as assuming both primary and secondary responses were observed. For the former case, the MMSE was iteratively determined by incorporating observations *y*_1*j*_; for the latter case, the MMSE was iteratively determined by incorporating pairs of observations (*y*_1*j*_, *y*_2*j*_). Confidence intervals were obtained from quantiles of the posterior distribution, which is defined by the posterior mean (the MMSE estimator) (31) and the posterior variance (32).

The MMSE prediction ${\hat{f}}_{1j^{*}}$ for individual #19 was iteratively determined after assuming only primary responses were observed and after assuming pairs of primary and secondary responses were observed. The iterative estimates for both cases are shown in Figure 5, along with the simulated data. The variance of the posterior distribution for *f*_1*j*^*∗*^_ from (32) was used to construct 95% confidence intervals on *f*_1*j*^*∗*^_. For the individual considered, the predictions based on both response variables (purple line) were more accurate than the predictions based on only the primary response (blue line) for the first few iterations of MMSE prediction. Note, however, that while this behavior of the prediction accuracy is found on average, as follows from (34) it is not necessarily true for each and every individual to which the procedure may be applied. Thus, caution is needed in relying on bivariate Bayesian forecasting for improved prediction accuracy of specific individuals; improved prediction accuracy can only be counted on in the average over individuals.

## 6. Data Collection Cost Minimization

When Bayesian forecasting is applied to individualize predictions, data must be collected to tailor the population model to the individual at hand. In certain sleep research applications, such as forecasting of sleep parameters across nights or predicting performance deficits across periods of sleep deprivation, and in a wide range of other biomedical contexts, this requires creating multiple opportunities for taking measurements. This may be an expensive proposition, and reducing the number of measurement bouts needed to obtain the necessary data could entail considerable cost savings. By measuring secondary responses and incorporating these through bivariate Bayesian forecasting, it may be possible to achieve a given level of prediction accuracy at lower overall cost of data acquisition. Here we explore this possibility in the case of the bivariate random intercept model.

We consider a scenario in which the cost of collecting an observation on the primary response is *c*_1_, the cost of collecting an observation on the secondary response is *c*_2_, and the total cost of data collection is the sum of primary and secondary response costs accrued,

(36) $$\begin{array}{c} c=m_{1}c_{1}+m_{2}c_{2}, \end{array}$$

where *m*_1_, *m*_2_ ≥ 0. For this scenario, we determine how many observations *m*_1_ and *m*_2_ we may expect to have to collect from each response type in order to minimize the total cost of achieving, on average, a given prediction accuracy *η* ^2^:

(37) $$\begin{array}{c} M_{{\hat{f}}_{1j^{*}}}=\eta^{2}. \end{array}$$

We can simplify the minimization problem by removing either *m*_1_ or *m*_2_ from both the total cost equation and the nonnegativity constraints on the number of data points. Using ${\hat{f}}_{1j^{*}}={\hat{\theta }}_{1}$ (see (30)), it follows that the BMSE of ${\hat{f}}_{1j^{*}}$ is equal to the BMSE of ${\hat{\theta }}_{1}$ (i.e., $M_{{\hat{f}}_{1j^{*}}}=M_{{\hat{\theta }}_{1}}$). Fixing $M_{{\hat{f}}_{1j^{*}}}=\eta^{2}$ (37) therefore implies that $M_{{\hat{\theta }}_{1}}=\eta^{2}$, which can be used with (34) to obtain a relationship between the number of primary and secondary observations needed to meet the average accuracy criterion *η* ^2^:

(38) $$\begin{array}{c} m_{1}=\sigma_{1}^{2}\left(\;\frac{1}{\eta^{2}}-\frac{1}{\delta_{1}^{2}}-\lambda \left(\;m_{2}\;\right)\;\right). \end{array}$$

We then substitute (38) into (36):

(39) $$\begin{array}{c} c=c_{1}\sigma_{1}^{2}\left(\;\frac{1}{\eta^{2}}-\frac{1}{\delta_{1}^{2}}-\lambda \left(\;m_{2}\;\right)\;\right)+c_{2}m_{2}. \end{array}$$

The constraint *m*_1_ ≥ 0 can be equivalently formulated as an upper bound on *λ*(*m*_2_):

(40) $$\begin{array}{c} \lambda \left(\;m_{2}\;\right)\leq \frac{1}{\eta^{2}}-\frac{1}{\delta_{1}^{2}}. \end{array}$$

Consideration of this constraint is only necessary if there are a certain number of measurements on the secondary response for which it is possible to obtain the desired accuracy without measurement of the primary response; that is,

(41) $$\begin{array}{c} \underset{m_{2}\to \infty }{\lim}\lambda \left(\;m_{2}\;\right)\geq \frac{1}{\eta^{2}}-\frac{1}{\delta_{1}^{2}}. \end{array}$$

Substituting for *λ*(*m*_2_) from (35), this latter condition can be reformulated as follows:

(42) $$\begin{array}{c} \delta_{1}^{2}\left(\;1-\rho^{2}\;\right)\leq \eta^{2}. \end{array}$$

Thus, *m*_2_ has an upper bound when *η* ^2^, the desired BMSE of ${\hat{f}}_{j^{*}}$, is not smaller than *δ*_1_^2^(1 − *ρ* ^2^), the minimum BMSE of ${\hat{f}}_{j^{*}}$ which can be obtained using only the secondary response. It follows that the constraint set for the minimization problem is

(43) 0≤m2≤σ221/η2−1/δ12δ221−ρ21/δ121−ρ2−1/η2if  δ121−ρ2≤η2,0≤m2otherwise.

If *m*_2_ exceeds its upper bound then the number of secondary response measurements is more than what is minimally needed to meet the average accuracy criterion (37).

**Figure 6:**
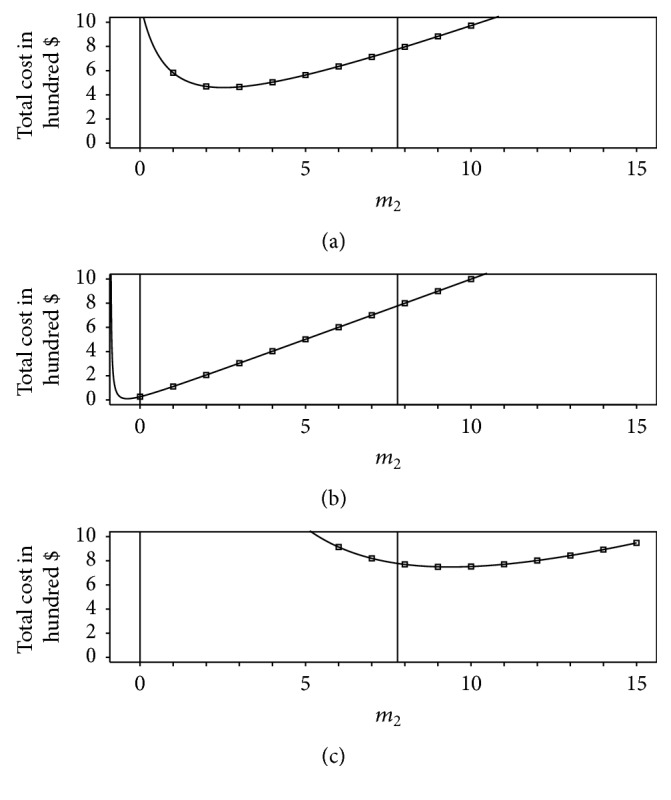
Illustration of three types of absolute minima of the total cost of obtaining a fixed average prediction accuracy inside the region defined by (43). Each plot shows the total cost *c* = *c*_1_ *m*_1_ + *c*_2_ *m*_2_ plotted against secondary response sample size *m*_2_. For *m*_2_ given, the primary response sample size *m*_1_ that obtains a fixed BMSE of *η* ^2^ in the expected primary response ${\hat{f}}_{j^{*}}$ is computed using (38). The lower and upper boundaries of the region defined by (43) are shown with solid vertical lines. (a) shows an interior point minimum, obtained by letting *σ*_1_ = 1.00. This type of solution occurs when the minimum of the unconstrained cost function lies within the region defined by (43). (b) shows a lower boundary solution, obtained by letting *σ*_1_ = 0.15. This type of solution occurs when the minimum of the unconstrained cost function lies below the feasible region defined by (43). (c) shows an upper boundary solution, obtained by letting *σ*_1_ = 3.00. This type of solution occurs when the minimum of the unconstrained cost function lies above the region defined by (43).

The minimal cost solution occurs either on the boundary of the region defined by (43) or at a local minimum in the interior of this region.  Figure 6 shows how different values of the error variance *σ* ^2^ can result in either boundary or interior solution types. For this demonstration, the population parameters are set at *ρ* = 0.85, *δ*_1_ = 1.0, *δ*_2_ = 1.0, and *σ*_2_ = 0.5, the costs of measurement are assumed to be *c*_1_ = $500 and *c*_2_ = $100, and the BMSE of ${\hat{f}}_{j^{*}}$ is fixed at the value *η* ^2^ = 0.30.

In cases where the solution lies in the interior of (43) at a local minimum, the solution must occur at critical points of (39), which can be found by setting to zero the derivative of the total cost with respect to *m*_2_:

(44) $$\begin{array}{c} \frac{\partial }{\partial m_{2}}c\left(\;m_{2}\;\right)=-c_{1}\sigma_{1}^{2}\frac{\partial \lambda \left(m_{2}\right)}{\partial m_{2}}+c_{2}=0, \end{array}$$

where

(45) $$\begin{array}{c} \frac{\partial \lambda \left(m_{2}\right)}{\partial m_{2}}=\frac{\sigma_{2}^{2}\rho^{2}\delta_{2}^{2}/\delta_{1}^{2}}{{\left(m_{2}\delta_{2}^{2}\left(1-\rho^{2}\right)+\sigma_{2}^{2}\right)}^{2}}. \end{array}$$

Substituting the above expression for ∂*λ*(*m*_2_)/∂*m*_2_ into (44) and solving for *m*_2_, we obtain the following two critical points:

(46) $$\begin{array}{c} m_{2}^{\pm }=\frac{\sigma_{2}}{\delta_{1}\delta_{2}^{2}\left(1-\rho^{2}\right)}\left(\;-\delta_{1}\sigma_{2}\pm \sqrt{\frac{c_{1}}{c_{2}}}\delta_{2}\sigma_{1}\left|\;\rho \;\right|\;\right). \end{array}$$

The smaller critical point *m*_2_^−^ can be disregarded as a possible solution since it is always less than zero. The second derivative at *m*_2_^+^,

(47) $$\begin{array}{c} \frac{\partial^{2}}{\partial m_{2}^{2}}c\left(\;m_{2}^{+}\;\right)=\frac{2c_{2}\sqrt{c_{2}/c_{1}}\left(1-\rho^{2}\right)\delta_{1}\delta_{2}}{\left|\rho \right|\sigma_{1}\sigma_{2}}, \end{array}$$

is positive when |*ρ* | <1, which implies that the cost function exhibits a local minimum at this point. If the local minimum *m*_2_^+^ is inside the region defined by (43) (see Figure 6(a)), then the solution to the cost minimization problem is

(48) m^1=σ121η2−1δ121−ρ21−c2δ12/σ12c1δ22/σ22ρ2,m^2=m2+,

where ${\hat{m}}_{1}$ is determined by substituting ${\hat{m}}_{2}$ into (38), and the total cost is found from (36).

Alternatively, if *m*_2_^+^ is below the lower boundary of the region defined by (43) (see Figure 6(b)), then the minimal cost solution involves collecting no data from the secondary response. The conditions for which the secondary response is not part of the minimal cost solution are as follows:

(49) $$\begin{array}{c} \frac{c_{2}}{c_{1}}>\frac{\delta_{2}^{2}/\sigma_{2}^{2}}{\delta_{1}^{2}/\sigma_{1}^{2}}\rho^{2}, \end{array}$$

where *δ*_*r*_^2^/*σ*_*r*_^2^ reflects the between-to-within variance ratio for the *r*th response type. For this case, the solution which achieves, on average, the level of accuracy *η* ^2^ is found from (38):

(50) m^1=σ121η2−1δ12,m^2=0.

Finally, if *m*_2_^+^ is above the upper boundary of the region defined by (43) (see Figure 6(c)), then the solution for *m*_2_ occurs at this boundary, where all the data are collected from the secondary response and none from the primary response. For this case, the solution is as follows:

(51) m^1=0,m^2=σ221/η2−1/δ12δ221−ρ21/δ121−ρ2−1/η2.

**Figure 7:**
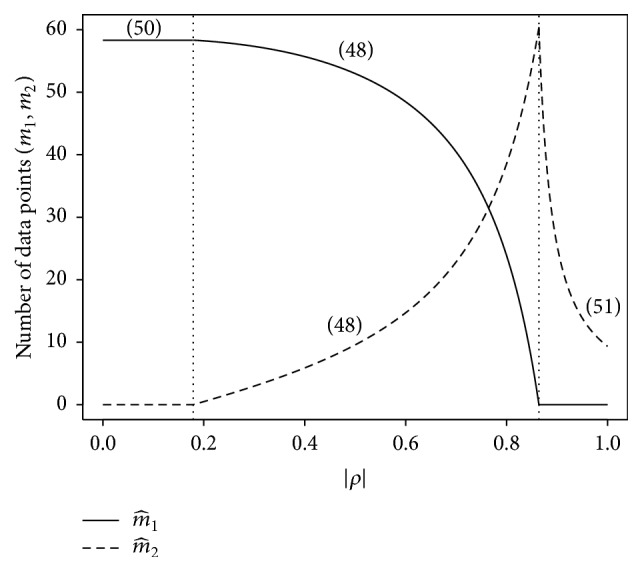
Example of data collection cost minimization that shows the number of observations on primary and secondary responses needed to obtain a fixed BMSE in the MMSE prediction of the expected primary response ${\hat{f}}_{j^{*}}$, for different values of the between-subjects correlation between primary and secondary response parameters. The solid curve represents the number of measurements to collect from the primary response, ${\hat{m}}_{1}$, and the dashed curve represents the number of measurements to collect from the secondary response, ${\hat{m}}_{2}$. In this example, for 0.0≤|*ρ* | ≤ 0.18, no data is to be collected from the secondary response, and the number of data points to collect from the primary response is obtained from (50). For 0.18<|*ρ* | ≤ 0.85, the number of secondary observations increases and the number of primary observations decreases with increasing correlation, as specified by ${\hat{m}}_{2}$ and ${\hat{m}}_{1}$ in (48). For 0.85<|*ρ* | ≤ 1.0, observations are to be collected only from the secondary response, the number of which is given by (51). The equation numbers are indicated near the relevant pieces of the curves.

Figure 7 illustrates the number of observations required from primary and secondary responses to obtain a fixed level of accuracy *η* ^2^ on average for different values of the between-subjects correlation *ρ* between primary and secondary response parameters. For the example shown, the population model parameters and cost parameters were fixed at *δ*_1_ = 1, *δ*_2_ = 1, *σ*_1_ = 1, *σ*_2_ = 0.5, *c*_1_ = 5, and *c*_2_ = 1, and the desired level of accuracy was *η* ^2^ = 0.30. The figure illustrates the three cases described by (48), (50), and (51).

## 7. Example: Efficient Assessment of an Individual's Characteristic Wakefulness after Sleep Onset

To illustrate the cost minimization approach outlined in the previous section, we apply it in an example involving the assessment of wakefulness after sleep onset (WASO) in laboratory-based sleep studies. Here we define WASO as the duration of intermittent wakefulness during a sleep period, between the time of sleep onset and the time of final awakening. WASO can be measured by polysomnography (PSG), that is, measuring the sleep electroencephalogram (EEG) and other physiological sleep signals and scoring sleep/wake states, typically in 30 s epochs, based on those signals. PSG is the gold standard procedure for sleep/wake assessment, but it is labor-intensive and expensive to perform. WASO may also be measured in the laboratory using wrist actigraphy (i.e., wrist activity monitoring), which is considerably less expensive. Although actigraphy is not considered a gold standard for measuring WASO, the correspondence with PSG-based WASO is at least moderate in healthy populations [24].

We base our example on data from *n* = 33 subjects (ages 22–38; 15 females) who spent between 6 and 13 nights and days inside a controlled laboratory environment with 10 h in bed for sleep (22:00–08:00) each day. The Institutional Review Board (IRB) of Washington State University approved the research, and subjects gave written informed consent. WASO was measured using both PSG (WASO-P) and actigraphy (WASO-A). PSG recordings were performed using digital equipment (Nihon Kohden, Foothill Ranch, CA). Sleep stages and periods of wakefulness were scored in 30 s epochs using standard criteria [25], and WASO-P was calculated from the scored records. Actigraphic recordings were made with Motionlogger wrist actigraphs (Ambulatory Monitoring, Inc., Ardsley, NY). Sleep and wakefulness were assessed from the actigraphic records using the automated algorithm of [26], which calculated WASO-A.

Let WASO-P be the primary response (as it is the gold standard measure) and let WASO-A be the secondary response. Assuming that WASO-P and WASO-A are normally distributed around distinct subject-specific means, we apply the model defined by (23)–(28) to our example. We anticipate that the subject-specific means for WASO-P and WASO-A are positively correlated. We aim to determine a cost-effective data collection scheme given a specific level of desired accuracy in estimates of an individual's mean WASO-P. For illustration purposes, we assume a fixed cost of $1,250 per night for WASO-P and $150 per night for WASO-A. We wish to estimate an individual's mean WASO-P to an average accuracy (i.e., $\sqrt{BMSE}$) of *η* = 15 min. Using the equations derived in the previous section, we estimate the number of nights of WASO-P and WASO-A that most cost-effectively achieves the desired level of accuracy on average.

We have estimated the population model parameters using the nlme package for R [27]. This package fits mixed-effects models using the approach of [28]. The maximum likelihood estimation method tends to underestimate variance parameters [29]; therefore, we estimate these parameters using the restricted maximum likelihood method [6].

In our dataset, the overall means for WASO-P and WASO-A were estimated as follows: *μ*_1_ = 54 min ± 5 min and *μ*_2_ = 32 min ± 4 min (estimate ± standard error), indicating that WASO-A tended to underestimate the total amount of WASO as compared to PSG. The estimated variability between subjects for WASO-P and WASO-A was found to be the same: *δ*_1_ = *δ*_2_ = 21 min. There was a substantial correlation between subject means for WASO-P and WASO-A: *ρ* = 0.69 (95% confidence interval: [0.30, 0.90]). The within-subject variation around the subject mean was *σ*_1_ = 32 min for WASO-P and *σ*_2_ = 21 min for WASO-A.

**Figure 8:**
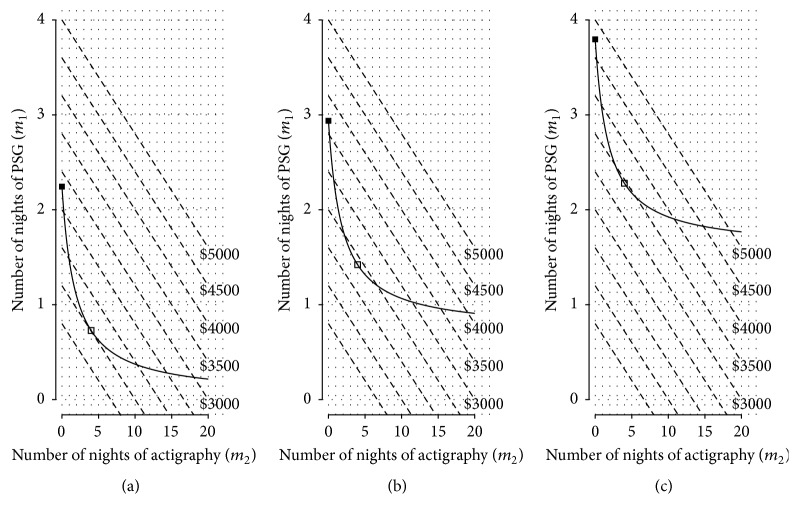
Cost of collection and number of nights of actigraphy and polysomnography (PSG) which combined will yield a certain average accuracy of wakefulness after sleep onset (WASO) parameter estimates. For illustration purposes, PSG and actigraphy are assumed to cost $1250 and $150 per night, respectively. The cost of data collection is fixed on each diagonal dashed line (for illustrative purposes these are only shown at fixed intervals) and increases as we collect more nights of PSG and actigraphy. Subject-specific WASO estimates of an average accuracy of (a) 15 min, (b) 14 min, and (c) 13 min are obtained on the solid curve. The point that minimizes the cost of obtaining the fixed accuracy (open squares) is determined as the point on the fixed accuracy curve where the line tangent to the curve is parallel to the fixed cost lines. For comparison the solution for obtaining the same average accuracy using only polysomnography is also shown (solid squares).

We determine from (43) that *m*_2_, the number of actigraphy nights, is not bounded above; that is, we cannot achieve our accuracy with actigraphy alone. Further, we find from (46) that *m*_2_^+^ is within the feasible region defined by (43), and, therefore, the solution to the cost minimization problem is given by (48). Applying these equations, we achieve an average accuracy of *η* = 15 min for minimal cost by collecting 3.99 nights of actigraphy and 0.73 nights of PSG (see Figure 8(a)). An approximately optimal solution in the integer domain is found by the common practice of rounding the optimal continuous solution to the nearest integer values [30]. We verify through a grid search that the minimal cost integer value solution neighbors the analytic solution and can be obtained by rounding down to three nights of actigraphy and up to one night of PSG. This yields a total cost of $1700. In contrast, to achieve the same accuracy with PSG alone would require 2.24, or in practice three nights of measurement, for a total cost of $3750.

Note that the results are highly dependent on the estimated between-subjects correlation and that the 95% confidence interval for this correlation was large. The minimal cost solution also depends on the level of accuracy that is required; see Figures 8(b) and 8(c) for scenarios with an average accuracy of 14 min and 13 min, respectively.

## 8. Linear Models with Time Dependency

For both the univariate and bivariate linear models, time dependency can be introduced by adding time as a covariate. This complicates the construction of a design matrix that enables predictions with a given average accuracy. We show that, in models with time dependency, the BMSE of a predicted response depends on the times at which responses are measured, and this dependency can be summarized by the mean and variance of the measurement times.

To illustrate, we consider a linear approximation of a time-dependent model known as the two-process model of sleep regulation [10, 31]. It has been shown that, for a range of sleep/wake scenarios, the two-process model can describe temporal changes in waking cognitive performance as the algebraic difference between two functions describing physiological processes: the homeostatic pressure for sleep and the circadian pressure for wakefulness [11]. Here we focus solely on modeling the homeostatic pressure for sleep, the dynamics of which are specified separately for sleep and for wakefulness. The dynamics can be modeled using the recursive formulation of [10]

(52) $$\begin{array}{c} S_{t}=\left\{\begin{array}{cc} e^{-\Delta t/\tau_{d}}S_{t-1} & \left(\text{sleep}\right)\\ 1-e^{-\Delta t/\tau_{r}}\left(1-S_{t-1}\right) & \left(\text{wake}\right), \end{array}\right. \end{array}$$

where *S*_*t*_ represents the homeostatic pressure after the *t*th time step of duration Δ*t*, *S*_*t*−1_ represents the homeostatic pressure at one time step before (i.e., at time (*t* − 1) · Δ*t*), Δ*t* is typically fixed at 0.5 h, and *τ*_*d*_ and *τ*_*r*_ are time constants for the decay and rise of the homeostatic process during sleep and wakefulness, respectively.

**Figure 9:**
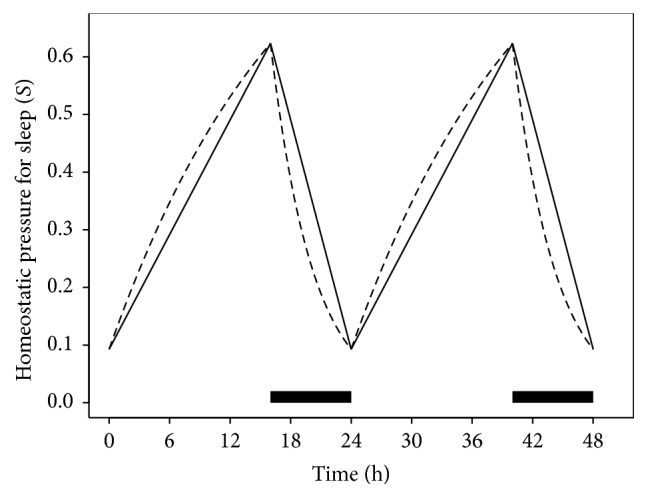
Homeostatic pressure for wakefulness plotted over two complete sleep/wake cycles for a repeating schedule with 16 h of wakefulness and 8 h time in bed for sleep in each cycle. The dashed line represents the homeostatic pressure for sleep as given by (52); the solid line is a linear approximation based on interpolation between the sleep/wake transition points. The parameter values *τ*_*d*_ = 4.2 h and *τ*_*r*_ = 18.2 h are taken from [10]. The initial condition (*S*_0_) is derived assuming steady state (54). Black bars indicate the 8 h periods in bed for sleep.

We divide the sleep/wake schedule into periods of sleep and periods of wake, both indexed by *k*. Let *S*_0_^(*k*)^ represent the initial homeostatic pressure for the *k*th period of wakefulness. It has been proposed that change over time in the model is better modeled as linear rather than exponential [32]. For the purpose of the present example, we adopt this idea and approximate the model over a period of continuous wakefulness using a linear interpolation between the start and end points of the wake period (see Figure 9).

Let *y*_*j*_ represent (hypothetical) measurements of the build-up of homeostatic pressure for sleep during wakefulness. These data can be modeled using the following approximation:

(53) $$\begin{array}{c} y_{j}=S_{0}^{\left(k\right)}+\frac{\left(1-e^{-T^{\left(k\right)}/\tau_{r}}\right)\left(1-S_{0}^{\left(k\right)}\right)}{T^{\left(k\right)}}t_{j}+\epsilon_{j}, \end{array}$$

where *t*_*j*_ represents the amount of time elapsed since awakening, *T* ^(*k*)^ is used to denote the duration of the *k*th wake period, and *ϵ*_*j*_ represents normally distributed measurement error.

We consider the homeostatic process over a repeating schedule consisting of *T* ^(*k*)^ = *T* = 16 h of wakefulness and 8 h of sleep. In this schedule, individuals maintain a steady state for which the homeostatic pressure *S*_0_^(*k*)^ at the onset of the wake period is constant across days; that is, *S*_0_^(*k*)^ = *S*_0_. This allows us to derive the homeostatic pressure at the start of wakefulness as a function of *τ*_*r*_ and *τ*_*d*_:

(54) $$\begin{array}{c} S_{0}=\frac{e^{-\left(24-T\right)/\tau_{d}}\left(1-e^{-T/\tau_{r}}\right)}{1-e^{-\left(\left(24-T\right)/\tau_{d}+T/\tau_{r}\right)}}. \end{array}$$

The equation for the homeostatic pressure during a particular wake period can thus be written as

(55) $$\begin{array}{c} y_{j}=\alpha +\beta t_{j}+\epsilon , \end{array}$$

where

(56) α=S0,

(57) β=1−e−T/τr1−S0T.

In matrix form, the model can be written as

(58) $$\begin{array}{c} y=H\theta +\epsilon , \end{array}$$

where the design matrix is given by

(59) $$\begin{array}{c} H=\left(\begin{array}{cc} 1 & t_{1}\\ \vdots & \vdots \\ 1 & t_{m} \end{array}\right), \end{array}$$

and the parameter vector is given by

(60) $$\begin{array}{c} \theta =\left(\begin{array}{c} \alpha \\ \beta \end{array}\right). \end{array}$$

Analogous to (5), but for two parameters, we assume the following prior distribution on model parameters:

(61) $$\begin{array}{c} \left(\begin{array}{c} \alpha \\ \beta \end{array}\right)\sim N\left(\;\mu ,\left(\begin{array}{cc} \delta_{\alpha }^{2} & 0\\ 0 & \delta_{\beta }^{2} \end{array}\right)\;\right). \end{array}$$

As in (6), we assume that the errors are independent realizations from a normal distribution with zero mean and variance *σ* ^2^. Analogous to (11) and (12), the BMSE for the MMSE prediction of the expected (primary) response ${\hat{f}}_{j^{*}}$ at some time *t*_*j*^*∗*^_ in the univariate case is as follows (see Appendix A):

(62) Mf^j∗=1/δβ2+ms2/σ2+mt¯−tj∗2/σ2+tj∗2/δα21/δα2+m/σ21/δβ2+ms2+t¯2/σ2−mt¯/σ22,

where $\bar{t}$ denotes the mean of these times,

(63) $$\begin{array}{c} \bar{t}=\frac{1}{m}\overset{m}{\underset{j=1}{\sum }}t_{j}, \end{array}$$

and *s* ^2^ denotes the variance of these times,

(64) $$\begin{array}{c} s^{2}=\frac{1}{m}\overset{m}{\underset{j=1}{\sum }}{\left(\;t_{j}-\bar{t}\;\right)}^{2}. \end{array}$$

Our task is to determine the measurement times *t*_1_,…, *t*_*m*_ for this example that will minimize $M_{{\hat{f}}_{j^{*}}}$. Equation (62) shows that $M_{{\hat{f}}_{j^{*}}}$ depends on the measurement times only through their mean $\bar{t}$ and variance *s* ^2^. Consequently, instead of conducting an *m*-dimensional minimization of $M_{{\hat{f}}_{j^{*}}}$ over all the measurement times *t*_1_,…, *t*_1*m*_, we can write $M_{{\hat{f}}_{j^{*}}}$ as a function of $\bar{t}$ and *s* ^2^ and conduct a two-dimensional minimization. In doing this, we find that $M_{{\hat{f}}_{j^{*}}}$ is minimized both when *s* ^2^ → *∞* (see Appendix B) and also, more practically relevant, when

(65) $$\begin{array}{c} \bar{t}=\frac{t_{j^{*}}\left(\sigma^{2}/m+\delta_{\alpha }^{2}\right)}{\delta_{\alpha }^{2}}. \end{array}$$

The optimal mean measurement time for this example, given by (65), lies slightly above the prediction time; in the limit as *δ*_*α*_^2^ → *∞*, $M_{{\hat{f}}_{j^{*}}}$ is minimized when the data are collected at times such that ${\bar{t}}_{min}=t_{j^{*}}$. If the prior variance on the intercept is equal to the error variance (i.e., *δ*_*α*_^2^ = *σ* ^2^), the effect of the Bayesian prior is equivalent to increasing the value of $\bar{t}$ that produces the minimum $M_{{\hat{f}}_{j^{*}}}$ by *t*_*j*^*∗*^_/*m*. This adjustment is the same as what would manifest with no prior information on the intercept when adding an additional measurement time, *t*_*m*+1_ = 0, to the design matrix **H**.

The absolute minimum of $M_{{\hat{f}}_{j^{*}}}$ in (62) is not always located in the feasible region in this example, as defined by 0 ≤ *t*_*j*_ ≤ *T*, ∀*j* ∈ {1,…, *m*}. More specifically, the absolute minimum of the unconstrained case is not located inside the feasible region if and only if (see Appendix B):

(66) $$\begin{array}{c} {\bar{t}}_{min}\geq T. \end{array}$$

Under this condition we hypothesize that, within the feasible region, $M_{{\hat{f}}_{j^{*}}}$ exhibits an absolute minimum when all the data are collected at time *T*. This is easy to show for the case of one measurement time (i.e., *m* = 1), and Appendix C contains a proof for the case of two measurement times (i.e., *m* = 2).

For *m* > 2, we conducted a simulation study to search for a counterexample (i.e., a case where the value of $M_{{\hat{f}}_{j^{*}}}$ when all data is collected at time *T* is not the smallest value of $M_{{\hat{f}}_{j^{*}}}$ within the feasible region). For the simulation study, we simulated 10,000,000 times with the following values:

(67) δα~Uniform0.001,1;δβ~Uniform0.001,1;σ~Uniform0.001,1;m~Discrete  Uniform1,2,…,100;tj∗~UniformTδα2σ2/m+δα2,T.

Concerning the ranges of the variance components, note that $M_{{\hat{f}}_{j^{*}}}$ is invariant to the scale of the response. This can be demonstrated by scaling the variance matrices **C**_*θ*_ and **C**_*ϵ*_ by *c*_*y*_ and showing that the resulting $M_{{\hat{f}}_{j^{*}}}$ is then scaled by the same factor *c*_*y*_. The conclusion is that the shape of the surface of $M_{{\hat{f}}_{j^{*}}}$ depends on the variance components only through their relative magnitudes. Furthermore, we argue that if any variance component is more than three orders of magnitude greater than any other component, then it would be advantageous to simplify the model by removing the smaller component. As such, all cases for which this model is reasonable can be covered within the range from 0.001 to 1 for each variance component. Further, concerning the number of observations, we expect that if there is a counterexample, it can be found somewhere in the range 1 ≤ *m* ≤ 100. Finally, the range for *t*_*j*^*∗*^_ is determined specifically so that the absolute minimum lies outside the feasible region.

For each simulation, we compared $M_{{\hat{f}}_{j^{*}}}$ at the hypothesized minimum, where *t*_*j*_ = *T*, ∀*j* ∈ {1,…, *m*}, to a randomly chosen time point, where each *t*_*j*_ is a realization from the following distribution:

(68) $$\begin{array}{c} t_{j}\sim \text{Uniform}\left(\;0,T\;\right). \end{array}$$

For each of the 10,000,000 simulations, $M_{{\hat{f}}_{j^{*}}}$ at the hypothesized minimum was indeed smaller than $M_{{\hat{f}}_{j^{*}}}$ at the randomly chosen time point. Thus, we found no evidence against our original hypothesis that $M_{{\hat{f}}_{j^{*}}}$ exhibits an absolute minimum if all the data are collected at time *T*. An analytical proof is beyond the scope of this paper.

We now extend our analysis to consider a time-dependent model with both primary and secondary responses. We formulate the model as follows:

(69) $$\begin{array}{c} y_{rj}=s_{rj}+\epsilon_{rj}=\alpha_{r}+\beta_{r}t_{rj}+\epsilon_{rj}. \end{array}$$

In matrix form, the model is

(70) $$\begin{array}{c} y=H\theta +\epsilon , \end{array}$$

where the design matrix is given by

(71) $$\begin{array}{c} H=\left(\begin{array}{cccc} 1 & 0 & t_{11} & 0\\ \vdots & \vdots & \vdots & \vdots \\ 1 & 0 & t_{1m_{1}} & 0\\ 0 & 1 & 0 & t_{21}\\ \vdots & \vdots & \vdots & \vdots \\ 0 & 1 & 0 & t_{2m_{2}} \end{array}\right), \end{array}$$

and the parameter vector is given by

(72) $$\begin{array}{c} \theta =\left(\begin{array}{c} \alpha_{1}\\ \alpha_{2}\\ \beta_{1}\\ \beta_{2} \end{array}\right). \end{array}$$

Let us assume the following prior distribution on model parameters:

(73) α1α2β1β2~Nμ,δα12ρδα1δα200ρδα1δα2δα220000δβ12ωδβ1δβ200ωδβ1δβ2δβ22.

As in (28), we assume that the errors are independent realizations from a normal distribution with zero mean and *σ*_*r*_  (*r* = 1,2). The BMSE of estimates of the primary response at time *t*_1*j*^*∗*^_ is given by

(74) $$\begin{array}{c} M_{{\hat{f}}_{1j^{*}}}=h^{\prime }M_{\hat{\theta }}h, \end{array}$$

where

(75) h=10t1j∗0,

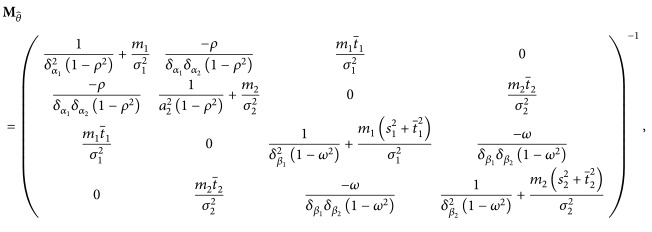
(76)where ${\bar{t}}_{1}$ denotes the mean of the primary measurement times,

(77) $$\begin{array}{c} {\bar{t}}_{1}=\frac{1}{m_{1}}\overset{m}{\underset{j=1}{\sum }}t_{1j}, \end{array}$$

${\bar{t}}_{2}$ denotes the mean of the secondary measurement times,

(78) $$\begin{array}{c} {\bar{t}}_{2}=\frac{1}{m_{2}}\overset{m}{\underset{j=1}{\sum }}t_{2j}, \end{array}$$

*s*_1_^2^ denotes the variance of the primary measurement times,

(79) $$\begin{array}{c} s_{1}^{2}=\frac{1}{m_{1}}\overset{m_{1}}{\underset{j=1}{\sum }}{\left(\;t_{1j}-{\bar{t}}_{1}\;\right)}^{2}, \end{array}$$

and *s*_2_^2^ denotes the variance of the secondary measurement times,

(80) $$\begin{array}{c} s_{2}^{2}=\frac{1}{m_{2}}\overset{m_{2}}{\underset{j=1}{\sum }}{\left(\;t_{2j}-{\bar{t}}_{2}\;\right)}^{2}. \end{array}$$

Our task in the bivariate case of the example is to determine the primary measurement times *t*_11_,…, *t*_1*m*_1__ and the secondary measurement times *t*_21_,…, *t*_2*m*_2__ that minimize $M_{{\hat{f}}_{1j^{*}}}$. Equations (74) and (76) show that $M_{{\hat{f}}_{1j^{*}}}$ depends on the measurement times only through their response-specific means and variances, ${\bar{t}}_{1}$, ${\bar{t}}_{2}$, *s*_1_^2^, and *s*_2_^2^. Consequently, it is sufficient to minimize $M_{{\hat{f}}_{1j^{*}}}$ with respect to ${\bar{t}}_{1}$, ${\bar{t}}_{2}$, *s*_1_^2^, and *s*_2_^2^ and choose any set of measurement times with these means and variances. We find that $M_{{\hat{f}}_{1j^{*}}}$ can be minimized by collecting data at the following times (see Appendix D):

(81) t¯1min=t1j∗δα121−ρ2δα22+σ22/m2+σ12/m1δα22+σ22/m2δα121−ρ2δα22+σ22/m2,

(82) t¯2min=0.

The optimal mean measurement time for the primary response variable, ${\bar{t}}_{1min}$ in (81), lies slightly above the prediction time. In the limit as *m*_2_ ↓ 0, the solution becomes the univariate solution:

(83) $$\begin{array}{c} {\bar{t}}_{1min}=\frac{t_{1j^{*}}\left(\delta_{\alpha_{1}}^{2}+\sigma_{1}^{2}/m_{1}\right)}{\delta_{\alpha_{1}}^{2}}. \end{array}$$

Again, the absolute minimum of $M_{{\hat{f}}_{1j^{*}}}$ is not always located in the feasible region defined by 0 ≤ *t*_*rj*_ ≤ *T*. More specifically, the absolute minimum of the unconstrained function is located outside of the feasible region if and only if (see Appendix D):

(84) $$\begin{array}{c} {\bar{t}}_{1min}\geq T. \end{array}$$

Under this condition, it seems logical that the minimum would occur if we were to collect all the primary data at time *T* and all the secondary data at time zero. However, a simulation study similar to that described above revealed counterexamples, which occurred when *ω* > 0.99. When *T* was decreased (or the ranges of the variance components for the slope were increased), counterexamples also occurred at smaller values of *ω*. Therefore, the optimal measurement scheme for the bivariate case appears to depend on *ω* and *T*. When *ω* is small or *T* is large (in comparison to the magnitudes of *δ*_*β*_1__ and *δ*_*β*_2__), the optimal design seems to be collecting all the primary data at *t* = *T* and all the secondary data at *t* = 0. When *ω* is large or *T* is small (compared to the magnitudes of *δ*_*β*_1__ and *δ*_*β*_2__), better designs are likely to be found numerically.

In summary, the average prediction accuracy for the simple linear time-dependent model depends not only on the number of measurements collected, but also on the times when these measurements are taken. In the univariate case, the prediction accuracy depends on these times only through their mean and variance. In the example of the two-process model, the optimal mean of the measurement times is slightly after the prediction time, where the delay increases with more prior information and with fewer or less informative data. When little prior information on the intercept is available, it is usually possible to collect data so that the absolute minimum of $M_{{\hat{f}}_{j^{*}}}$ is achieved. In the case where the theoretical minimum cannot be achieved (i.e., (66) is not satisfied), minimization of $M_{{\hat{f}}_{j^{*}}}$ can be achieved by collecting all the data at time *T*.

In the bivariate case of our example, the prediction accuracy depends on the measurement times only through the means and variances of the primary and secondary measurement times. Furthermore, $M_{{\hat{f}}_{1j^{*}}}$ is minimized by centering the primary measurement times above *t*_1*j*^*∗*^_ (see (81)) as in the univariate case and collecting all secondary measurements at time zero. As in the univariate case, when little prior information on the primary intercept is available, it is usually possible to collect data so that the absolute minimum of $M_{{\hat{f}}_{1j^{*}}}$ is achieved. In the case where this minimum cannot be achieved (i.e., (84) is not satisfied), minimization of $M_{{\hat{f}}_{1j^{*}}}$ can usually be obtained for parameter ranges considered in our simulation by collecting all primary data at time *T*, and all secondary data at time zero.

## 9. Nonlinear Models with Time Dependency

Lastly, we focus briefly on the nonlinear case, where the BMSE generally lacks a closed form solution. Obtaining the BMSE of the MMSE estimator for nonlinear models typically requires numerical integration of the joint probability density of **y** and ***θ***. We illustrate this with an example in which we numerically estimate the prediction BMSE given a nonlinear model and a single primary response measurement and show how it can be improved by a secondary response measurement.

We consider a two-parameter sinusoidal model of circadian (i.e., 24 h) rhythmicity, defined for a given subject and a bivariate response (*r* = 1,2), as follows:

(85) $$\begin{array}{c} y_{rj}=f_{rj}+\epsilon_{rj}=A_{r}\sin\left(\;\frac{2\pi \left(t_{rj}-\phi \right)}{24}\;\right)+\epsilon_{rj}, \end{array}$$

where *t* is in hours; *A*_*r*_ represents a response-specific amplitude; and *ϕ* represents the phase, which is assumed to be common to both response types. For our example, we assume

(86) A1A2~N55,10.950.951,ϕ~N0,2.

We simulated *n* = 1000 individuals from this model using normally distributed errors, with primary and secondary response standard deviations of *σ*_1_ = 0.25 and *σ*_2_ = 0.1. For each individual, we simulated a single primary response at time *t*_11_ = 14 and a single secondary response at time *t*_12_ = 22. Bayesian forecasting was performed using Markov Chain Monte Carlo (MCMC) with a chain length of 100,000 to obtain the MMSE predictions *f*_1*j*^*∗*^_ for each individual at time *t*_1*j*^*∗*^_ = 24. Predictions were constructed using a primary data point alone, and also using both a primary data point and a secondary data point.

**Figure 10:**
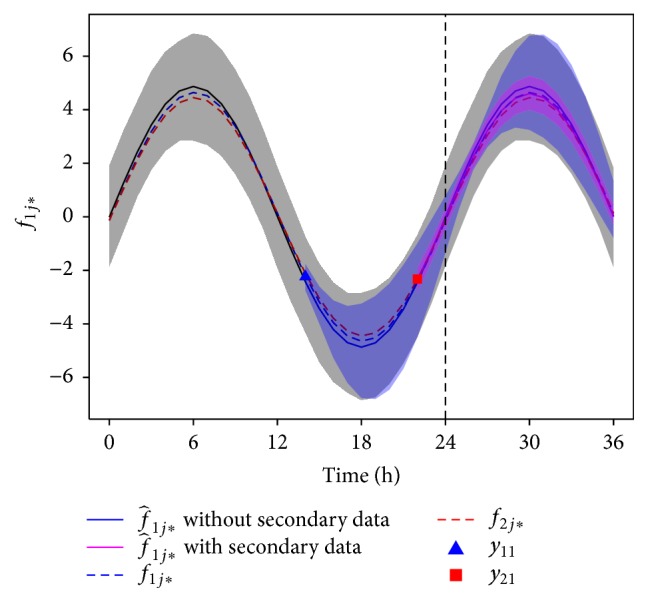
MMSE predictions of the expected primary response ${\hat{f}}_{1j^{*}}$ and corresponding 95% confidence intervals for a randomly simulated individual from the model specified in (85). The MMSE estimator ${\hat{f}}_{1j^{*}}$ is determined assuming each of the following: no data was observed (black line and gray confidence interval), a single primary response was observed (blue line and confidence interval), and both a primary and a secondary response were observed (purple line and confidence interval). The expected primary and secondary responses using the subject's simulated parameter values are shown with blue and red dashed lines. The black vertical dashed line shows the time at which predictions are made.

Figure 10 shows these predictions for a randomly chosen individual. The 95% confidence intervals on *f*_1*j*^*∗*^_ were constructed using the quantiles of the posterior distribution for *f*_1*j*^*∗*^_. For the individual considered, the predictions based on both response variables (purple line and shading) are at many times substantially more accurate (i.e., they have smaller posterior variance) than the predictions based on only the primary response (blue line and shading).

The average accuracy over individuals was assessed at time *t*_1*j*^*∗*^_ = 24 by estimating the BMSE, as follows (cf. (3)):

(87) $$\begin{array}{c} {\hat{M}}_{{\hat{f}}_{1ij^{*}}}=\frac{1}{N}\overset{N}{\underset{i=1}{\sum }}{\left(\;f_{1ij^{*}}-{\hat{f}}_{1ij^{*}}\;\right)}^{2}. \end{array}$$

The estimated BMSE of ${\hat{f}}_{1ij^{*}}$ when using only the primary data points was 0.53, and the estimated BMSE of ${\hat{f}}_{1ij^{*}}$ when using both the primary and secondary data points was 0.20. These results suggest that there are nonlinear modeling scenarios where secondary response variables can improve predictions of primary response variables considerably via between-subjects correlation.

## 10. Discussion

In this paper, we illustrated in a Bayesian, repeated-measures framework how to improve the prediction accuracy of the expected response, as measured by the BMSE, for a primary response variable in the presence of a secondary response variable that is correlated with the primary response variable between subjects. To set up the general procedure of improving prediction accuracy through Bayesian forecasting, we constructed the BMSE for a simple univariate random intercept model.

We applied the general procedure to a bivariate random intercept model and derived the BMSE of the primary response predictions for this model. We studied how the BMSE depends on the number of observations from the secondary response and found that, for a fixed number of primary response observations, the BMSE is bounded below as specified by (40). The potential value of considering a secondary response may be assessed by considering whether this lower bound represents a meaningful improvement in the expected prediction accuracy.

Assuming the availability of a reasonably highly correlated secondary variable we also addressed the problem of determining the number of primary and secondary measurements needed to obtain a given average accuracy at minimal cost. We derived equations for the solution to this problem and illustrated their use with an example from sleep research. Given previously observed means, variances, and between-subjects correlation for polysomnographic (primary) and actigraphic (secondary) measurements of wakefulness after sleep onset and assumed reasonable measurement costs, we found that, to obtain an average prediction accuracy of 15 min, the use of actigraphy in addition to polysomnography resulted in a substantial reduction in estimated data collection costs as compared to polysomnography alone.

We then considered a steady-state, linear approximation of the homeostatic component for the two-process model of sleep regulation [10, 31] in the univariate case. We found that the minimization of the BMSE with respect to the vector of times at which the data are collected can be divided into two subcases. In one subcase, the BMSE is minimized by collecting data at times with a mean value slightly above the time at which predictions are to be made, with the offset being inversely proportional to the variance of the prior on the intercept. In the second subcase, all of the data is to be collected at the maximal time point. We extended the results of this example to the bivariate case, which again can be divided into two subcases. In one subcase, the BMSE is minimized by collecting the data so that the secondary measurement times have a mean of zero, and the primary variable is collected at times with a mean value somewhat above the time at which the predictions are to be made, like in the univariate case. In the other subcase, the time points that minimize the BMSE may best be found numerically.

Finally, we considered a nonlinear circadian model and determined the improvement in individualized prediction accuracy from a single primary data point versus both a single primary data point and a single secondary data point. For this particular model (85), we found the improvement to be substantial, suggesting that there are cases for nonlinear models where a secondary variable can substantially improve prediction accuracy for the primary variable, given a reasonably high between-subjects correlation.

In conclusion, depending on the between- and within-subjects variance components and the between-subjects correlation between primary and secondary responses, using secondary response data can be effective in increasing the individualized prediction accuracy on the primary response variable in Bayesian forecasting.

This work represents an improvement over the work of Chandler and colleagues [19], who proposed incorporating secondary variables in individualized performance predictions as covariates in a generalized linear model. An advantage of their approach is that it accounts for perturbations on system dynamics from external factors that are common to the outcome variables considered. A drawback is that the approach does not accumulate information about individual differences over time and therefore does not become increasingly accurate for individualized predictions as more data are collected for the individual at hand. Furthermore, the technique requires secondary data to be measured at the same time as the primary data is to be predicted. This is not a requirement for the presently proposed method, for which individualized predictions can be made for any given time, even if secondary data are unavailable then.

That said, here we considered only Bayesian models with diagonally structured error covariance matrices. Such models do not account for correlation within subjects between response types. The work of Chandler and colleagues [19] does account for such correlation, using a fixed linear relationship between primary and secondary responses. We did not consider this possibility here, using merely a diagonal error covariance matrix with one parameter for each response type. However, the Bayesian modeling framework in this paper can be expanded to account for correlation within subjects between response types by adding additional structure to the error covariance matrix.

Finally, the models described here assume that error variance is constant over repeated measures and across different subjects. This constraint can be relaxed easily by allowing the error covariance matrix to have different elements for different individuals.

The multivariate repeated-measures Bayesian forecasting framework presented here may be useful in a variety of clinical settings. One example is modeling the disabling effects of chronic back pain, where pain-related fear may be a good choice for a secondary variable [33]. For other examples, a rich literature in this area can be found in the domain of anesthesiology [34], where clinical applications of multivariate Bayesian forecasting abound.

## Appendices

### A. Expression for the Univariate Linear Time-Dependent Model

Theorem A.1 A.1. The BMSE of MMSE predictions for prediction BMSE in the univariate, linear, time-dependent model given by (58) is given by

(A.1) $$\begin{array}{c} M_{{\hat{f}}_{j^{*}}}=h^{\prime }{\left(\;C_{\theta }^{-1}+H^{\prime }C_{\epsilon }^{-1}H\;\right)}^{-1}h, \end{array}$$

where

(A.2) $$\begin{array}{c} h=\left(\begin{array}{c} 1\\ t_{j^{*}} \end{array}\right), \end{array}$$

where *t*_*j*^*∗*^_ represents the time for which the expected response is predicted, **C**_*θ*_ is the prior variance matrix for the parameter vector ***θ***, **H** is the design matrix, **C**_*ϵ*_ is the error covariance matrix, and $M_{{\hat{f}}_{j^{*}}}$ is defined in (3).

Proof Since

(A.3) $$\begin{array}{c} f_{j^{*}}=h^{\prime }\theta \end{array}$$

and since the MMSE estimator commutes over linear transformations [23], the MMSE estimator of the expected response is given by

(A.4) $$\begin{array}{c} {\hat{f}}_{j^{*}}=h^{\prime }\hat{\theta }. \end{array}$$

Therefore,

(A.5) Mf^j∗Efj∗−f^j∗2=Efj∗−f^j∗fj∗−f^j∗′=Eh′θ−θ^θ−θ^′h=h′Eθ−θ^θ−θ^′h=h′Mθ^h,

where the parameter BMSE matrix $M_{\hat{\theta }}$ is given by [23] as follows:

(A.6) $$\begin{array}{c} M_{\hat{\theta }}\equiv E\left[\;\left(\;\theta -\hat{\theta }\;\right){\left(\;\theta -\hat{\theta }\;\right)}^{\prime }\;\right]={\left(\;C_{\theta }^{-1}+H^{\prime }C_{\epsilon }^{-1}H\;\right)}^{-1}. \end{array}$$

Therefore, the BMSE of the estimated response is given by (A.1).

Theorem A.2 A.2. The BMSE of MMSE predictions for the univariate, linear, time-dependent model given by (58) is given by

(A.7) Mf^j∗=1/δβ2+ms2/σ2+mt¯−tj∗2/σ2+tj∗2/δα21/δα2+m/σ21/δβ2+ms2/σ2+mt¯2/δα2σ2,

where $M_{{\hat{f}}_{j^{*}}}$ is given in (A.1).

Proof For this model, the inverse of the between-subjects covariance matrix is given by

(A.8) $$\begin{array}{c} C_{\theta }^{-1}=\left(\begin{array}{cc} \frac{1}{\delta_{\alpha }^{2}} & 0\\ 0 & \frac{1}{\delta_{\beta }^{2}} \end{array}\right). \end{array}$$

Furthermore, the parameter BMSE matrix is given by (see Theorem A.1)

(A.9) $$\begin{array}{c} M_{{\hat{f}}_{j^{*}}}=h^{\prime }{\left(\;C_{\theta }^{-1}+H^{\prime }C_{\epsilon }^{-1}H\;\right)}^{-1}h, \end{array}$$

where **h** is the covariate vector at the time at which we want to make predictions:

(A.10) $$\begin{array}{c} h=\left(\begin{array}{c} 1\\ t_{j^{*}} \end{array}\right). \end{array}$$

The error variance matrix is as follows:

(A.11) $$\begin{array}{c} C_{\epsilon }=\sigma^{2}I. \end{array}$$

The design matrix is as follows:

(A.12) $$\begin{array}{c} H=\left(\begin{array}{cc} 1 & t_{1}\\ \vdots & \vdots \\ 1 & t_{m} \end{array}\right). \end{array}$$

The matrix multiplication **H**′**C**_*ϵ*_^−1^ **H** can be computed to be

(A.13) $$\begin{array}{c} H^{\prime }C_{\epsilon }^{-1}H=\frac{1}{\sigma^{2}}\left(\begin{array}{cc} m & \overset{m}{\underset{j=1}{\sum }}t_{j}\\ \overset{m}{\underset{j=1}{\sum }}t_{j} & \overset{m}{\underset{j=1}{\sum }}t_{j}^{2} \end{array}\right). \end{array}$$

The matrix inverse (**C**_*θ*_^−1^ + **H**′**C**_*ϵ*_^−1^ **H**)^−1^ can then be computed as follows:

(A.14) Cθ−1+H′Cϵ−1H−1=1δα2+mσ2∑j=1mtjσ2∑j=1mtjσ21δβ2+∑j=1mtj2σ2−1=1δα2+mσ21δβ2+∑j=1mtj2σ2−∑j=1mtjσ22−1·1δβ2+∑j=1mtj2σ2−∑j=1mtjσ2−∑j=1mtjσ21δα2+mσ2.

Computing the quadratic form, we find that

(A.15) $$\begin{array}{c} M_{{\hat{f}}_{j^{*}}}=\frac{1/\delta_{\beta }^{2}+\left(\sum_{j=1}^{m}t_{j}^{2}\right)/\sigma^{2}-\left(2t_{j^{*}}\sum_{j=1}^{m}t_{j}\right)/\sigma^{2}+t_{j^{*}}^{2}\left(1/\delta_{\alpha }^{2}+m/\sigma^{2}\right)}{\left(1/\delta_{\alpha }^{2}+m/\sigma^{2}\right)\left(1/\delta_{\beta }^{2}+\left(\sum_{j=1}^{m}t_{j}^{2}\right)/\sigma^{2}\right)-{\left(\left(\sum_{j=1}^{m}t_{j}\right)/\sigma^{2}\right)}^{2}}. \end{array}$$

We note the following decomposition of the sum of squared times:

(A.16) ∑j=1mtj2∑j=1mtj−t¯+t¯2=∑j=1mtj−t¯2+∑j=1mt¯2=ms2+t¯2.

Including this decomposition, we find that

(A.17) Mf^j∗=1/δβ2+ms2/σ2+mt¯2/σ2−2mtj∗t¯/σ2+tj∗2/δα2+mtj∗2/σ21/δα2+m/σ21/δβ2+ms2+t¯2/σ2−mt¯/σ22=1/δβ2+ms2/σ2+m/σ2t¯2−2tj∗t¯+tj∗2+tj∗2/δα21/δα2+m/σ21/δβ2+ms2+t¯2/σ2−mt¯/σ22=1/δβ2+ms2/σ2+mt¯−tj∗2/σ2+tj∗2/δα21/δα2+m/σ21/δβ2+ms2/σ2+mt¯2/δα2σ2.

### B. Unconstrained Minimization of the BMSE for the Univariate, Time-Dependent Linear Model

Theorem B.1 B.1. For the univariate, linear, time-dependent model given by (58), $M_{{\hat{f}}_{j^{*}}}$ as given by (A.17) exhibits an absolute minimum at a point $\left({\bar{t}}_{min},s_{min}^{2}\right)$ if and only if

(B.1) $$\begin{array}{c} M_{{\hat{f}}_{j^{*}}}=\frac{1}{1/\delta_{\alpha }^{2}+m/\sigma^{2}}. \end{array}$$

Proof Recall that $\bar{t}$ and *s* ^2^ are defined by (63) and (64). To prevent ambiguous notation, let *γ* = *s* ^2^. Consider the derivative of $M_{{\hat{f}}_{j^{*}}}$ with respect to *γ*:

(B.2) ∂Mf^j∗t¯,γ∂γ=−mσ2tj∗σ2+m−t¯+tj∗δα22δβ4mδα2σ2+mγδβ2+σ2σ2+mγ+t¯2δβ22.

We can represent this derivative more simply as follows:

(B.3) $$\begin{array}{c} \frac{\partial M_{{\hat{f}}_{j^{*}}}\left(\bar{t},\gamma \right)}{\partial \gamma }=-\frac{m\sigma^{2}c_{1}^{2}\delta_{\beta }^{4}}{c_{2}^{2}}, \end{array}$$

where the values of *c*_1_ and *c*_2_ are found explicitly from (B.2). It is easy to show that *c*_1_^2^ ≥ 0 and *c*_2_^2^ > 0, and consequently

(B.4) $$\begin{array}{c} \frac{\partial M_{{\hat{f}}_{j^{*}}}\left(\bar{t},\gamma \right)}{\partial \gamma }\leq 0. \end{array}$$

Therefore, for a fixed $\bar{t}$, $M_{{\hat{f}}_{j^{*}}}$ either decreases or remains constant as we increase *γ*. It follows that, for fixed value of $\bar{t}$, $M_{{\hat{f}}_{j^{*}}}$ is bounded below by $M_{{\hat{f}}_{j^{*}}}$ in the limit as *γ* → *∞*:

(B.5) $$\begin{array}{c} \underset{\gamma \to \infty }{\lim}M_{{\hat{f}}_{j^{*}}}\left(\;\bar{t},\gamma \;\right)=\frac{1}{1/\delta_{\alpha }^{2}+m/\sigma^{2}}. \end{array}$$

Since the limit of $M_{{\hat{f}}_{j^{*}}}$ does not depend on the values of $\bar{t}$ and *γ*, any values of $\bar{t}$ and *γ* for which $M_{{\hat{f}}_{j^{*}}}$ takes on the value given by (B.5) are an absolute minimum of $M_{{\hat{f}}_{j^{*}}}$.

Theorem B.2 B.2. For the univariate, linear, time-dependent model given by (58), an absolute minimum of $M_{{\hat{f}}_{j^{*}}}$ as given by (A.17), inside the region defined by 0 ≤ *t*_*j*_ ≤ *T*, can be obtained by collecting data at times *t*_*j*_ such that

(B.6) $$\begin{array}{c} \bar{t}=\frac{t_{j^{*}}\left(\sigma^{2}/m+\delta_{\alpha }^{2}\right)}{\delta_{\alpha }^{2}}, \end{array}$$

if and only if

(B.7) $$\begin{array}{c} \bar{t}\leq T. \end{array}$$

Proof The critical points of the unconstrained function are found by taking the derivative of **M**_*f*_*j*^*∗*^__ with respect to $\bar{t}$ and *s* ^2^ and setting these derivatives to zero. The derivative with respect to $\bar{t}$ is as follows:

(B.8) $$\begin{array}{c} \frac{\partial M_{{\hat{f}}_{j^{*}}}\left(\bar{t},s^{2}\right)}{\partial \bar{t}}=-\frac{2m\sigma^{2}\left(t_{j^{*}}\sigma^{2}+m\left(-\bar{t}+t_{j^{*}}\right)\delta_{\alpha }^{2}\right)\delta_{\beta }^{2}\left(\bar{t}t_{j^{*}}\sigma^{2}\delta_{\beta }^{2}+\delta_{\alpha }^{2}\left(\sigma^{2}+ms^{2}\delta_{\beta }^{2}\right)\right)}{{\left(m\delta_{\alpha }^{2}\left(\sigma^{2}+ms^{2}\delta_{\beta }^{2}\right)+\sigma^{2}\left(\sigma^{2}+m\left(s^{2}+{\bar{t}}^{2}\right)\delta_{\beta }^{2}\right)\right)}^{2}}. \end{array}$$

The derivative with respect to *s* ^2^ is given by (B.2). Setting both derivatives equal to zero, we find the following critical point:

(B.9) $$\begin{array}{c} {\bar{t}}_{min}=\frac{t_{j^{*}}\left(\sigma^{2}/m+\delta_{\alpha }^{2}\right)}{\delta_{\alpha }^{2}}. \end{array}$$

Evaluating the BMSE at this critical point, we find that

(B.10) $$\begin{array}{c} M_{{\hat{f}}_{j^{*}}}\left(\;{\bar{t}}_{min},s^{2}\;\right)=\frac{1}{1/\delta_{\alpha }^{2}+m/\sigma^{2}}, \end{array}$$

which is sufficient to show that $M_{{\hat{f}}_{j^{*}}}$ exhibits an absolute minimum at this point. Given the constraint that 0 ≤ *t*_*j*_ ≤ *T* for each *j*, ${\bar{t}}_{min}$ is a feasible point only when $0\leq {\bar{t}}_{min}\leq T$. It is easy to see that ${\bar{t}}_{min}>0$ in all cases. Therefore, the absolute minimum can be achieved by collecting all data at ${\bar{t}}_{min}$ if and only if ${\bar{t}}_{min}\leq T$.

### C. Constrained Minimization of the BMSE for the Univariate, Time-Dependent Linear Model with Two Time Points

Theorem C.1 C.1. For the univariate, linear, time-dependent model given by (58) with *m* = 2 and 0 ≤ *t*_*j*_ ≤ *T*, if

(C.1) $$\begin{array}{c} \frac{t_{j^{*}}\left(\sigma^{2}/2+\delta_{\alpha }^{2}\right)}{\delta_{\alpha }^{2}}>T, \end{array}$$

then $M_{{\hat{f}}_{j^{*}}}$ as given by (A.17) is minimized by collecting data at times such that *t*_1_ = *t*_2_ = *T*.

Proof Substituting *m* = 2 into (A.15) we find that

(C.2) $$\begin{array}{c} M_{{\hat{f}}_{j^{*}}}=\frac{\left(t_{1}^{2}+t_{2}^{2}\right)/\sigma^{2}-\left(2\left(t_{1}+t_{2}\right)t_{j^{*}}\right)/\sigma^{2}+t_{j^{*}}^{2}\left(\left(2/\sigma^{2}\right)+\left(1/\delta_{\alpha }^{2}\right)\right)+\left(1/\delta_{\beta }^{2}\right)}{-{\left(t_{1}+t_{2}\right)}^{2}/\sigma^{4}+\left(\left(2/\sigma^{2}\right)+\left(1/\delta_{\alpha }^{2}\right)\right)\left(\left(t_{1}^{2}+t_{2}^{2}\right)/\sigma^{2}+\left(1/\delta_{\beta }^{2}\right)\right)}. \end{array}$$

From Theorem B.2 we know that there are no critical points inside the feasible region, and the solution must therefore exist on the boundary of the region, which is defined by the following line segments:

(C.3) A:  t1∈0,T,t2=0;B:  t1=0,t2∈0,T;C:  t1∈0,T,t2=T;D:  t1=T,t2∈0,T.

Note that $M_{{\hat{f}}_{j^{*}}}$ is symmetric in *t*_*j*_:

(C.4) $$\begin{array}{c} M_{{\hat{f}}_{j^{*}}}\left(\;t_{1}=a,\;\;t_{2}=b\;\right)=M_{{\hat{f}}_{j^{*}}}\left(\;t_{1}=b,\;\;t_{2}=a\;\right). \end{array}$$

Therefore, it is sufficient to consider only line segments *A* and *C*. We first consider finding the minimum value on line segment *A*. Setting *t*_2_ = 0 and then taking the derivative of $M_{{\hat{f}}_{j^{*}}}$ with respect to *t*_1_, and solving for *t*_1_, we find the following two critical points:

(C.5) t1minA+=tj∗σ2+2δα2δα2,

(C.6) t1minA−=−σ2δα2tj∗σ2+δα2δβ2.

Applying condition (C.1) to *t*_1min_^*A*+^, we find that *t*_1min_^*A*+^ > 2*T*. Taking the second derivative of $M_{{\hat{f}}_{j^{*}}}$ with respect to *t*_1_, such that *t*_2_ = 0, and evaluating it at *t*_1min_^*A*+^ result in

(C.7) ∂2Mf^j∗∂t12=2σ2δα8δβ2σ2+2δα22σ4tj∗2δβ2+3σ2tj∗2δα2δβ2+δα4σ2+2tj∗2δβ2,

which is positive. Therefore (C.5) represents a minimum. It is easy to see that *t*_1min_^*A*−^ is always negative. Taking the second derivative of $M_{{\hat{f}}_{j^{*}}}$ with respect to *t*_1_, such that *t*_2_ = 0, and evaluating it at *t*_1min_^*A*−^ (C.6) result in

(C.8) ∂2Mf^j∗∂t12=−2tn4σ2+δα22δβ6σ6tn2δβ2+3σ4tn2δα2δβ2+δα4σ4+2σ2tn2δβ2,

which is negative. Therefore *t*_1min_^*A*−^ represents a maximum. Since the maximum occurs at a value *t*_1min_^*A*−^ < 0, the minimum occurs at a value *t*_1min_^*A*+^ > *T*, and these are the only two critical points; the BMSE must be decreasing over the line segment, and the minimum BMSE on the line segment therefore occurs at *t*_1_ = *T*, *t*_2_ = 0. We next consider finding the minimum value on the line segment *C*. Setting *t*_2_ = *T* and then taking the derivative of $M_{{\hat{f}}_{j^{*}}}$ with respect to *t*_1_, and solving for *t*_1_, we find the following two critical points:

(C.9) t1minC+=σ2tj∗−Tδα2+2tj∗δα2δα2,t1minC−=δα2σ2+T2δβ2−Ttj∗δβ2−σ2tj∗+Tδα2−tj∗δα2δβ2.

We see that *t*_1min_^*C*+^ is minimized by applying the lower bound for *t*_*j*^*∗*^_ given by (C.1), and we find that *t*_1min_^*C*+^ > *T*. Concerning *t*_1min_^*C*−^, the numerator can be minimized by applying the upper bound for *t*_*j*^*∗*^_ of *T*. In this case, the numerator is *δ*_*α*_^2^ *σ* ^2^, which is always positive. The maximum value of the denominator is found by substituting in the minimum value for *t*_*j*^*∗*^_ given by (C.1). We find that the maximum value is −*Tσ* ^2^ *δ*_*α*_^2^ *δ*_*β*_^2^/(*σ* ^2^ + 2*δ*_*α*_^2^), which is negative. Therefore *t*_1min_^*C*−^ is always negative. Taking the second derivative of $M_{{\hat{f}}_{j^{*}}}$ with respect to *t*_1_, such that *t*_2_ = *T*, and evaluating it at *t*_1min_^*C*+^ result in

(C.10) $$\begin{array}{c} \frac{\partial^{2}M_{{\hat{f}}_{j^{*}}}}{\partial t_{1}^{2}}=\frac{2\sigma^{2}\delta_{\alpha }^{8}\delta_{\beta }^{2}}{{\left(\sigma^{2}+2\delta_{\alpha }^{2}\right)}^{2}\left(\sigma^{4}t_{j^{*}}^{2}\delta_{\beta }^{2}+\sigma^{2}t_{j^{*}}\left(-2t_{f}+3t_{j^{*}}\right)\delta_{\alpha }^{2}\delta_{\beta }^{2}+\delta_{\alpha }^{4}\left(\sigma^{2}+2t_{f}^{2}\delta_{\beta }^{2}-4t_{f}t_{j^{*}}\delta_{\beta }^{2}+2t_{j^{*}}^{2}\delta_{\beta }^{2}\right)\right)}. \end{array}$$

Evaluating (C.10) at *t*_*j*^*∗*^_ = *T* results in

(C.11) $$\begin{array}{c} \frac{\partial^{2}M_{{\hat{f}}_{j^{*}}}}{\partial t_{1}^{2}}=\frac{2\delta_{\alpha }^{8}\delta_{\beta }^{2}}{{\left(\sigma^{2}+2\delta_{\alpha }^{2}\right)}^{2}\left(\delta_{\alpha }^{4}+\sigma^{2}t_{f}^{2}\delta_{\beta }^{2}+t_{f}^{2}\delta_{\alpha }^{2}\delta_{\beta }^{2}\right)}, \end{array}$$

which is positive. In general, (C.10) is positive when

(C.12) σ4tj∗2δβ2+σ2tj∗−2tf+3tj∗δα2δβ2+δα4σ2+2tf2δβ2−4tftj∗δβ2+2tj∗2δβ2>0.

Setting the expression on the left-hand side to zero and solving for *t*_*j*^*∗*^_, we find the following roots:

(C.13) $$\begin{array}{c} t_{j^{*}}=\frac{\sigma^{2}t_{f}\delta_{\alpha }^{2}\delta_{\beta }^{2}+2t_{f}\delta_{\alpha }^{4}\delta_{\beta }^{2}\pm \sqrt{-\sigma^{6}\delta_{\alpha }^{4}\delta_{\beta }^{2}-3\sigma^{4}\delta_{\alpha }^{6}\delta_{\beta }^{2}-2\sigma^{2}\delta_{\alpha }^{8}\delta_{\beta }^{2}-\sigma^{4}t_{f}^{2}\delta_{\alpha }^{4}\delta_{\beta }^{4}-2\sigma^{2}t_{f}^{2}\delta_{\alpha }^{6}\delta_{\beta }^{4}}}{\sigma^{4}\delta_{\beta }^{2}+3\sigma^{2}\delta_{\alpha }^{2}\delta_{\beta }^{2}+2\delta_{\alpha }^{4}\delta_{\beta }^{2}}. \end{array}$$

These roots are imaginary, which implies that (C.10) is always positive, and therefore, *t*_1min_^*C*+^ is a minimum. Given that the BMSE must always be positive, and there are only two critical points, this implies that *t*_1min_^*C*−^ is a maximum. Since the maximum occurs at a value of *t*_1min_^*C*−^ < 0 and the minimum occurs at a value of *t*_1min_^*C*+^ > *T*, the minimum on the line segment must occur at the value *t*_1_ = *T*. In summary, the minimum value on the line segment *A* occurs at *t*_1_ = *T*, *t*_2_ = 0. Applying symmetry, the BMSE at this point is the same as the BMSE at the points *t*_1_ = 0, *t*_2_ = *T*, which is the maximum point for the line segment *C*. Therefore, the minimum BMSE does not occur on the line segment *A*. Again, by symmetry, this means that the minimum BMSE does not occur on the line segment *B*. For the line segment *C*, the minimum BMSE occurs at *t*_1_ = *T*, *t*_2_ = *T*. Applying symmetry, the minimum BMSE on the line segment *D* occurs at *t*_1_ = *T*, *t*_2_ = *T*. Since these two points are the same, we find that the overall minimum BMSE within the feasible region occurs at the point *t*_1_ = *t*_2_ = *T*.

### D. Unconstrained Minimization of the BMSE for the Bivariate, Time-Dependent Linear Model

Theorem D.1 D.1. For the bivariate, linear, time-dependent model given by (70), $M_{{\hat{f}}_{1j^{*}}}$ as given by (74) exhibits an absolute minimum at a point $\left({\bar{t}}_{1min},{\bar{t}}_{2min},s_{1min}^{2},s_{2min}^{2}\right)$ if and only if

(D.1) Mf^1j∗=δα12σ12/m11−ρ2δα22+σ22/m2σ12/m1δα22+σ22/m2+a121−ρ2δα22+σ22/m2.

Proof Recall that ${\bar{t}}_{1}$, ${\bar{t}}_{2}$, *s*_1_^2^, and *s*_2_^2^ are defined in (77), (78), (79), and (80). To prevent ambiguous notation, let *γ*_1_ = *s*_1_^2^ and *γ*_2_ = *s*_2_^2^. Consider the derivative of $M_{{\hat{f}}_{1j^{*}}}$ (see (74)) with respect to *γ*_1_:

(D.2) $$\begin{array}{c} \frac{\partial M_{{\hat{f}}_{1j^{*}}}\left({\bar{t}}_{1},{\bar{t}}_{2},\gamma_{1},\gamma_{2}\right)}{\partial \gamma_{1}}=-\frac{\delta_{\beta_{1}}^{2}m_{1}\sigma_{1}^{2}c_{1}^{2}}{c_{2}^{2}}, \end{array}$$

where

(D.3) c1=t¯2ρωδα1δα2δβ2m2σ12σ22+tj∗δβ1σ12δα22m2s22−1+ω2δβ22m2−σ22−σ22−s22+t¯22−1+ω2δβ22m2+σ22+t¯1−tj∗δα12δβ1m1−1+ρ2δα22m2s22−1+ω2·δβ22m2−σ22+σ22−s22+t¯22−1+ω2δβ22m2+σ22,c2=−2t¯1t¯2ρωδα1δα2δβ1δβ2m1m2σ12σ22+σ12δα22m2−s12+t¯12·δβ12m1s22−1+ω2δβ22m2−σ22+σ12s22δβ22m2+σ22+σ22−s12+t¯12·δβ12m1s22+t¯22−1+ω2δβ22m2−σ22+σ12s22+t¯22δβ22m2+σ22+a12m1−1+ρ2δα22m2s12δβ12m1s22−1+ω2δβ22m2−σ22−σ12s22δβ22m2+σ22+σ22σ12s22+t¯22δβ22m2+σ22+s12δβ12m1−s22+t¯22−1+ω2δβ22m2+σ22.

It is easy to show that *c*_1_^2^ ≥ 0 and *c*_2_^2^ ≥ 0, and consequently

(D.4) $$\begin{array}{c} \frac{\partial M_{{\hat{f}}_{1j^{*}}}\left({\bar{t}}_{1},{\bar{t}}_{2},\gamma_{1},\gamma_{2}\right)}{\partial \gamma_{1}}\leq 0. \end{array}$$

Consider also the derivative of $M_{{\hat{f}}_{1j^{*}}}$ with respect to *γ*_2_:

(D.5) $$\begin{array}{c} \frac{M_{{\hat{f}}_{1j^{*}}}\left({\bar{t}}_{1},{\bar{t}}_{2},\gamma_{1},\gamma_{2}\right)}{\partial \gamma_{2}}=-\frac{\delta_{\beta_{2}}^{2}m_{2}\sigma_{1}^{4}\sigma_{2}^{2}c_{3}^{2}}{c_{4}^{2}}, \end{array}$$

where

(D.6) c3=t¯2ρδα1δα2δβ2m2s12+t¯1t¯1−tj∗−1+ω2·δβ12m1−σ12+t¯1−tj∗ωδα12δβ1m1−1+ρ2·δα22m2−σ22+tj∗ωδβ1σ12δα22m2+σ22,c4=−2t¯1t¯2ρωδα1δα2δβ1δβ2m1m2σ12σ22+σ12δα22m2−s12+t¯12·δβ12m1s22−1+ω2δβ22m2−σ22+σ12s22δβ22m2+σ22+σ22−s12+t¯12·δβ12m1s22+t¯22−1+ω2δβ22m2−σ22+σ12s22+t¯22δβ22m2+σ22+a12m1−1+ρ2δα22m2s12δβ12m1s22−1+ω2δβ22m2−σ22−σ12s22δβ22m2+σ22+σ22σ12s22+t¯22δβ22m2+σ22+s12δβ12m1−s22+t¯22−1+ω2δβ22m2+σ22.

It can be shown that *c*_3_^2^ ≥ 0 and *c*_4_^2^ > 0, and consequently

(D.7) $$\begin{array}{c} \frac{\partial M_{{\hat{f}}_{1j^{*}}}\left({\bar{t}}_{1},{\bar{t}}_{2},\gamma_{1},\gamma_{2}\right)}{\partial \gamma_{2}}\leq 0. \end{array}$$

Therefore, for fixed ${\bar{t}}_{1}$ and ${\bar{t}}_{2}$, $M_{{\hat{f}}_{1j^{*}}}$ either decreases or remains constant as we increase *γ*_1_ or *γ*_2_. It follows that, for fixed values of ${\bar{t}}_{1}$ and ${\bar{t}}_{2}$, $M_{{\hat{f}}_{1j^{*}}}$ is bounded below by $M_{{\hat{f}}_{1j^{*}}}$ in the limit as *γ*_1_, *γ*_2_ → *∞*:

(D.8) limγ1,γ2→∞⁡Mf^1j∗t¯1,t¯2,γ1,γ2=δα12σ12/m11−ρ2δα22+σ22/m2σ12/m1δα22+σ22/m2+a121−ρ2δα22+σ22/m2.

Since the limit of $M_{{\hat{f}}_{1j^{*}}}$ does not depend on ${\bar{t}}_{1}$ or ${\bar{t}}_{2}$, any values of ${\bar{t}}_{1},{\bar{t}}_{2},\gamma_{1}$, and *γ*_2_ for which $M_{{\hat{f}}_{1j^{*}}}$ takes on the value given by (D.8) are an absolute minimum of $M_{{\hat{f}}_{1j^{*}}}$.

Theorem D.2 D.2. For the bivariate, linear, time-dependent model given by (70), an absolute minimum of $M_{{\hat{f}}_{1j^{*}}}$ as given by (74), inside the region defined by 0 ≤ *t*_*rj*_ ≤ *T*, can be obtained by collecting data at times *t*_*rj*_ such that

(D.9) t¯1min=t1j∗δα121−ρ2δα22+σ22/m2+σ12/m1δα22+σ22/m2δα121−ρ2δα22+σ22/m2,t¯2min=0,

if and only if

(D.10) $$\begin{array}{c} {\bar{t}}_{1min}\leq T. \end{array}$$

Proof Substituting (D.9) into $M_{{\hat{f}}_{1j^{*}}}$, we find that

(D.11) Mf^1j∗t¯1min,t¯2min,s12,s22=δα12σ12/m11−ρ2δα22+σ22/m2σ12/m1δα22+σ22/m2+a121−ρ2δα22+σ22/m2,

which is sufficient to show that any point $\left({\bar{t}}_{1min},{\bar{t}}_{2min},s_{1}^{2},s_{2}^{2}\right)$ is an absolute minimum of the unconstrained function (see Theorem D.1). Given the constraint that 0 ≤ *t*_1*j*_ ≤ *T* for each *j*, ${\bar{t}}_{1min}$ is a feasible point only when $0\leq {\bar{t}}_{1min}\leq T$. It is easy to see that ${\bar{t}}_{1min}>0$ in all cases. Therefore, the absolute minimum can be achieved by collecting all data at ${\bar{t}}_{1min}$ if and only if ${\bar{t}}_{1min}\leq T$.
